# Age-related change in inhibitory processes when controlling working memory capacity and processing speed: A confirmatory factor analysis

**DOI:** 10.1371/journal.pone.0316347

**Published:** 2025-01-27

**Authors:** Nuria Carriedo, Odir A. Rodríguez-Villagra, Juan A. Moriano, Pedro R. Montoro, Valentín Iglesias-Sarmiento

**Affiliations:** 1 Departamento de Psicología Evolutiva y de la Educación, National Distance Education University (UNED), Madrid, Spain; 2 Institute for Psychological Research, University of Costa Rica, San José, Costa Rica; 3 Cognitive Neuroscience Department, Neuroscience Research Center, University of Costa Rica, San José, Costa Rica; 4 Departamento de Psicología Social y de las Organizaciones, National Distance Education University (UNED), Madrid, Spain; 5 Departamento de Psicología Básica I, National Distance Education University (UNED), Madrid, Spain; 6 Departamento de Psicología Evolutiva y Comunicación, Campus Universitario de Vigo, University of Vigo, Vigo, Spain; University of Porto Faculty of Psychology and Educational Sciences: Universidade do Porto Faculdade de Psicologia e de Ciencias da Educacao, PORTUGAL

## Abstract

The main purpose of this study was to examine the age-related changes in inhibitory control of 450 children at the ages of 7–8, 11–12, and 14–16 when controlling for working memory capacity (WMC) and processing speed to determine whether inhibition is an independent factor far beyond its possible reliance on the other two factors. This examination is important for several reasons. First, empirical evidence about age-related changes of inhibitory control is controversial. Second, there are no studies that explore the organization of inhibitory functions by controlling for the influence of processing speed and WMC in these age groups. Third, the construct of inhibition has been questioned in recent research. Multigroup confirmatory analyses suggested that inhibition can be organized as a one-dimension factor in which processing speed and WMC modulate the variability of some inhibition tasks. The partial reliance of inhibitory processes on processing speed and WMC demonstrates that the inhibition factor partially explains the variance of inhibitory tasks even when WMC and processing speed are controlled and some methodological concerns are addressed.

## Introduction

Cognitive development entails processing efficiency and capacity changes, which refers to encompassing general-purpose changes in the ability to process and maintain information simultaneously (working memory capacity; WMC hereafter). Furthermore, it entails processing speed, inhibitory control, and their dynamic interrelations [1–3]. Although WMC, processing speed, and inhibitory control contribute differently, they support each other to achieve adaptative behavior [1–4].

However, theoretical perspectives differ in the importance attributed to specific factors as drivers of cognitive development. Inhibitory processes—the ability to focus attention on relevant information while filtering out irrelevant information or responses—have been emphasized as crucial for processing efficiency [5, 6]. In this view, inhibition is seen as the primary explanatory mechanism for cognitive development [7, 8] and for developmental changes in executive functioning [7]. The Inhibitory Deficit Theory also highlights the key role of inhibition [9, 10], proposing that reduced inhibitory control contributes to the decline in WM performance typically observed with aging.

Further, neo-Piagetian theories [11, 12] and the executive attention view of WMC [13–16] have also underlined the role of WMC as a domain-general mechanism that explains individual differences in cognitive development and complex cognition in general. Additionally, processing speed has been associated with age-related cognitive changes throughout the life-span [17]. Processing speed has been found to limit executive functioning, specifically, WMC [1, 18] and inhibitory control [19, 20].

However, despite the importance of inhibitory processes for cognitive development, empirical evidence about the age-related changes and organization of inhibitory control is scarce and controversial. Furthermore, to the best of our knowledge, there are no studies that explore the organization of inhibitory control across development by controlling the influence of processing speed and WMC in these age groups. Additionally, some authors have recently questioned whether inhibition should be considered a separate construct from processing speed [21].

To fill these research gaps, the main purpose of this study was to examine the organization of inhibitory processes during school ages of 7–8, 11–12, and 14–16 (7, 11, and 15 years old so far), given the critical importance of inhibitory control in cognitive development, developmental disorders [5–8], and developmental changes in executive functioning [7].

Given that our research focused on middle and late childhood, these ages were selected because main spurts occur in brain growth (weight and connectivity) in correlation with the main Piagetian stages of cognitive development [22, 23] and main changes in executive functioning [24] during this developmental period. Although we focused in this age groups, executive functions begin to develop during preechool with the ability to manage conflict during information processing being a critical skill in the development of executive functioning at this period [25–28].

The study also aimed to explore the structure of inhibitory control when controlling other essential factors in cognitive development, such as processing speed and WMC, and thus, to determine whether inhibition is an independent factor far beyond its possible reliance on processing speed and WMC.

### The construct of inhibition and its relation to WMC and processing speed

Despite the important role of inhibition in cognition and cognitive development, its definition has always been controversial because inhibition does not seem to be a unitary construct. Instead, the term “inhibition” encompasses different processes examined under different experimental paradigms, making it difficult to compare them. Thus, for some authors, the best way of conceptualizing inhibition is to consider it a family of processes, functionally and developmentally distinct [29, 30]. In the arena of cognitive development and aging research, several taxonomies have been proposed to classify inhibitory processes under different dimensions [6, 8, 29, 31, 32]. The definition of inhibitory functions and conceptual correspondence among different taxonomies can be seen in Table 1.

**Table 1:** Inhibitory functions and correspondence between different taxonomies about inhibitory processes.

Inhibitory functions	Definitions	Correspondence among conceptual taxonomies					
		Friedman & Miyake* [30]	Dempster [29]	Harnishfeger [6]	Nigg [8]	Hasher et al. [9]	Diamond [31]
***Resistance to distractor interference***	Ability to resist or resolve interference from information in the external environment irrelevant to the ongoing task. It occurs at the initial stage of processing, where relevant information must be selected, and the irrelevant must be ignored.	Resistance to distractor interference	Control of perceptual interference		Interference control	Access function	Attentional inhibition
***Prepotent response inhibition***	Ability to suppress dominant, automatic, or prepotent responses deliberately. This corresponds to a later stage of processing in which appropriate responses must be selected and incorrect ones inhibited.	Prepotent response inhibition	Control of motor interference	Behavioral inhibition	Behavioral inhibition Oculomotor inhibition	Restraint function	Behavioral inhibition
***Cognitive inhibition***	Ability to resist memory intrusions from relevant information that has become irrelevant in the ongoing task. It occurs at the middle stage of processing.	Resistance to proactive interference	Control of verbal interference	Cognitive inhibition	Cognitive inhibition	Deletion function	Inhibition of mental representations

Note: Friedman and Miyake [30] tried to integrate the different conceptual distinctions or taxonomies, postulating that these conceptual distinctions correspond to different stages of information processing.

Additionally, Munakata et al. [33] postulated a unified framework for inhibitory control distinguishing two main types of inhibition subserved by, at least, two different types of neural mechanisms that can work in concert: (1) directed global inhibition of subcortical and archicortical regions by pre-frontal cortex, and (2) indirect competitive inhibition within cortical and subcortical regions. Directed global inhibition helps to cope with stressors by inhibiting responses and suppressing memory retrieval. Indirect competitive inhibition provides top-down support for strengthening the most active representations while suppressing competitors. For instance, in the Stroop task, this involves enhancing color representation while inhibiting alternative representations (such as word meaning).

Therefore, the development of inhibitory processes cannot be fully understood without considering inhibition’s potential reliance on WMC, which is viewed as a limited-capacity system for temporarily maintaining and processing information [34–36]. Furthermore, the ability to keep relevant information active while suppressing distractions is recognized as central to WMC [37]. Consequently, WMC is also regarded as the strength of the executive attention system [38], which is responsible for maintaining and retrieving relevant information under conditions of interference as maintained by the executive attention view of WMC [10, 14–16, 39–41].

Thus, it is broadly assumed that efficient performance on inhibitory tasks requires keeping the task’s goal in mind to differentiate between relevant and irrelevant information, thus decreasing the probability of committing inhibitory errors [25, 33, 42, 43]. For instance, Munakata et al. [43] proposed that goal representations maintained in working memory provide top-down support through excitatory connections from the prefrontal cortex to activate appropriate representations. These representations compete with others via inhibitory connections, highlighting the importance of goal representation in overcoming conflict.

In line with previous research highlighting the reciprocal support between inhibition and working memory capacity [2, 44], some neuropsychological studies in adults also identified a shared network for WM and interference [45, 46]. This neural overlapping may explain the involvement of inhibitory processes in WMC tasks and the implication of WMC processes in inhibitory tasks, as well as a joint mechanism between the two processes [46].

Regarding the relationship between inhibition and processing speed, which is defined as the “ability to control attention to automatically, quickly, and fluently perform relatively simple repetitive cognitive tasks” [47, p.148] it has been proposed that individual differences in the effectiveness of higher-order cognitive abilities are driven by processing speed and that during infancy, processing speed is the ability underlying individual differences and predicting executive functions for later stages of cognitive development [48]. Moreover, other studies have also demonstrated that individual and developmental differences in inhibitory control were due to differences in processing speed rather than response inhibition or interference control [20, 46, 49–52]. On the contrary, some empirical evidence supports the view that differences in processing speed do not fully account for age-related changes in response inhibition [53, 54]. Luna et al. [2] also reported that processing speed allows efficient WMC, but not response inhibition in adolescents.

Therefore, processing speed, WMC, and inhibitory control primarily developed independently, but they work concurrently and interactively in the cognitive control of behavior to assist optimally adaptive behavior during cognitive development supported by overlapping but distinct neuronal mechanisms [2, 55].

### Factor structure of inhibitory functions

In the cognitive development framework, abundant empirical evidence is available on the development of the different inhibitory functions (response inhibition, cognitive inhibition, or resistance to distractor interference) but not on the interactions among these different inhibitory functions across development, that is, on age-related changes in their structure and organization.

The studies that focused on the development of the single inhibitory functions have demonstrated the progressive development in efficiency, consistency, and task independence of the different inhibitory processes from early childhood, over the elementary school years, to adulthood, reaching stabilization in adolescence or sometimes in adulthood [6, 31, 53, 56–60]. However, as it is argued, the most dramatic developmental changes are seen not in the ability to inhibit but in the consistency of successful inhibition during the task [6, 61]. Although younger children can inhibit inappropriate responses very early in development [62], consistent inhibiting requires goal-oriented top-down processes that orchestrate sensory and cognitive demands, requiring executive organization and control [42].

In contrast to the huge number of studies on the development of single inhibitory functions, studies addressing the factor structure of inhibition were conducted only in the last decades. In a seminal work with young adults, Friedman and Miyake [30] examined the relations among the three types of inhibition-related functions―*resistance to distractor interference*, *prepotent response inhibition*, and *resistance to proactive interference*―using the latent variable approach on a sample of young adults. It was found that *prepotent response inhibition* and *resistance to distractor interference* were closely related (*r* = .68) despite their previous conceptual distinction. Thus, they began to be considered as one construct: *response-distractor inhibition*, which was not related to *resistance to proactive* interference. This overlapping between distractor interference and response inhibition was corroborated in young adults both at the behavioral and neuropsychological [63] levels [64]. Comparatively, some other studies did not find this overlap [21, 65] or that cognitive inhibition and *response-distractor inhibition* measured the same construct [66].

In the developmental arena, we found only a few studies about the structure and organization of inhibitory functions. Gandolfi et al. [67], in a study with young children between 24 and 48 months, found a single (undifferentiated) inhibition factor in 2-year-olds that evolved through a two-factor model in 3-year-olds, where *resistance to interference* and *response inhibition* were already dissociated. This dissociation was corroborated in 65 children aged 5 and 6 years [68]. Moreover, using principal component analysis, Zamora et al. [69] reported three independent components–*resistance to interference*, *response inhibition*, and *cognitive inhibition* in 435 children (8 to 12-year-olds).

Regarding the factor structure of inhibitory control and WMC in children, we found only two studies in the literature. Shing et al. [70] conducted one of them with 263 participants aged 4 to 14 years ―divided into three groups of younger (4―6.7-year-olds), middle (6.8―9.5-year-olds), and oldest (9.5―14.6-year-olds) ― in which two correlated factors were identified through confirmatory factor analysis: one factor was for inhibitory control (comprising *response inhibition* and *resistance to interference* indicators) and another was for memory maintenance. An increasing differentiation after 9–10 years was also seen between memory maintenance and inhibitory control driven by inhibition. On its part, Tiego et al. [71], from a study conducted on 136 pre-adolescents aged 11–12 years, proposed a hierarchical model of two independent low-order factors (response inhibition and attentional inhibition) dependent on WMC.

### The present study

This study aimed to examine cross-sectionally the factorial structure of inhibitory functions at three age groups: 7-, 11-, and 15-year-olds. As a starting point, we adopted the conceptual distinctions of resistance to distractor interference, prepotent response inhibition, and cognitive inhibition (see Table 1).

Moreover, for theoretical and methodological reasons, we controlled both WMC and processing speed. Theoretically, individual and group differences in WMC and processing speed could moderately facilitate or hinder inhibition. Particularly, in young children, a lower processing speed could reduce WMC [3] and WMC could easily become overwhelmed and, consequently, make it harder to inhibit [2, 72]. Methodologically, most of the tasks used to tap into inhibitory processes are speed-based measures that take reaction time (RT) as the dependent variable. Thus, perceptual speed could primarily influence RT measures in inhibitory functioning tasks [50, 73]. Therefore, one possibility is that the differentiation between the constructs of *response-distractor inhibition*―tapped by time-dependent tasks―and *cognitive inhibition*―tapped mainly by accuracy tasks―could be primarily due to differences in processing speed and not due to different types of inhibitory processes [30, 50]. Moreover, most authors agree on the reliance of response to distractor interference and inhibition of prepotent responses on active goal maintenance or WMC [30, 33, 43, 44, 71, 74].

According to our objectives, we have three main research questions:

1) *What is the factor structure of the inhibition-related processes*, *and how does it progress from 7 to 15 years*? Studies conducted with children seem to indicate the differentiation between response inhibition and resistance to distractors as early as the age of three [67–69]. In contrast, studies conducted with young adults showed a single undifferentiated factor of response-distractor inhibition differentiated from cognitive inhibition [30]. This pattern of age-related changes is challenging to conciliate with the differentiation hypothesis proposed in related fields as the development of abstract intelligence [75] and executive functioning [54, 76, 77], and those that could also be expected for inhibitory control.

However, comparing studies conducted with children and adults just mentioned is complicated because of the diverse methodological issues: different tasks and low correlations among them, dependent variables, criteria to consider the independence of the factors, selection of models, and trimming procedures. For instance, the seemingly contradictory findings on the differentiation between resistance to distractors and response inhibition in children versus adults can often be attributed to these methodological issues. This is particularly evident when comparing the study by Gandolfi et al. [67] with children and Friedman and Miyake [30] study with young adults. Gandolfi et al. [67] identified two distinct constructs––response inhibition and resistance to distractors––in 3-year-olds, despite a high correlation (*r* = 0.71), indicating significant overlap. In contrast, Friedman and Miyake [30] combined these constructs in young adults, based on a correlation of .68.

Similarly, other methodological differences make it challenging to compare studies with children. The results found by Traverso et al. [68] were difficult to compare because they used formative indicators rather than reflective ones ones (see [78] for a discussion about formative and reflective indicators of the latent construct of EF). In Zamora et al.’s [79] study, only one task per hypothetical inhibitory process was included, making it hard to distinguish task-specific variance from inhibition-related processes.

Given these concerns about previous developmental evidence about the factor structure of inhibitory control, but consistent with other developmental evidence [6–8, 31] and the differentiation hypothesis, we expected a progressive differentiation of inhibitory factors until adolescence (H1).

2) *To what extent is the factor structure of the inhibition-related process the same when controlling for WMC and processing speed*, *and how does it progress from 7 to 15 years*? Consistent with previous evidence [2, 3, 53], we predicted that WMC, processing speed, and inhibitory control, although related, are separated factors. Therefore, we predicted that although the structure of the inhibition-related process could change when controlling for WMC and processing speed, we will still find significant factorial loadings of indicators of inhibitory tasks on inhibitory factor or factors (H2).

3) *Does the contribution of WMC and processing speed in inhibitory tasks performance vary across ages*? In agreement with previous research, we expected that WMC and processing speed do not contribute equally to inhibitory processes at different ages. Specifically, a lower processing speed in young children could reduce WMC [3]. Moreover, given the cognitive system’s limited capacity, WMC could easily become overwhelmed, making it harder to inhibit [2, 31, 72]. Instead, the higher processing efficiency and WMC of older children would support inhibitory control because efficient processing could increase the probability of holding information in WMC and then decrease the probability of committing inhibitory errors [31] (H3).

## Materials and methods

The data were collected as part of a more extensive, multifaceted study. Individuals participated in four separate sessions, lasting four to eight weeks. They completed 12 different executive tasks and took other psychometric tests to measure fluid intelligence, reading comprehension, and academic aptitudes. We included only data from inhibitory and WMC tasks. The testing began in October 2012 and ended in January 2013.

This extensive data collection is part of a larger research program aimed at addressing various research questions and objectives related to the development of executive functions (EF) and their relationship with academic achievement. The data presented in this paper have not been published before concerning the development and organization of inhibitory processes.

### Participants

The sample for this study included 450 volunteer students. Their distribution by age was as follows: 7-year-olds (150: 75 girls and 75 boys; age range 7.2–8.3, M = 7.3, SD = .42); 11-year-olds (150: 77 girls and 73 boys; range 10.10–12.3, M = 11, SD = .40); and 15-year-olds (150: 84 girls and 66 boys; range 14.11–16.1, M = 15, SD = .40). Participants were recruited from seven middle-class primary and secondary urban schools. The average family salary was slightly below the national average. None of the children was at risk of poverty or had any history of neurological impairment or developmental disabilities, according to educational and clinical reports provided by Guidance Departments of schools. The parents were requested to provide written informed consent before testing began. The Ethical Committee of the University approved the study in January 25, 2011.

### Tasks

Several criteria guided the task selection. The tasks (1) were well established to measure the construct addressed; (2) showed evidence of robust age-related changes; and (3) were correctly understood by all the participants. In all RT tasks, visual and auditory cues were provided to help participants remain active, maintain, and remember the task rules. A mapping for response keys, which did not compete with the ongoing stimuli, was constantly provided in all the computer tasks at the bottom of the computer screen to reduce WM load. We conducted multiple pilot studies with a different sample to (a) guarantee the task’s understandability (especially to younger children), (b) calibrate the stimuli presentation times, (c) ensure age-related differences among age groups, and (d) avoid floor or ceiling effects. All tasks included practice trials with feedback before the experimental ones. Tasks administered were the same for the age groups except for the receptive attention task—a standardized test—in which the stimuli for 7-year-olds differed from those for 11- and 15-year-olds.

In Table 2, you can find a brief description of the task. A complete description is available in the Supplementary Materials (Section A).

**Table 2:** Task description.

Inhibitory Constructs	Tasks	Description
Resistance to distractor interference	Flanker [79]	We used a modified version of the classical task [26, 80], where children were shown a row of five fish, with a central target fish surrounded by two flanker fish on each side, facing either right or left. The flanker fish could either point in the same direction as the target (congruent condition) or in the opposite direction (incongruent condition). In each trial, the children were instructed to identify the direction of the central fish (block 1) or the direction of the flanker fish (block 2) or alternate their attention between the central and flanker fish (block 3) by pressing a key to flanker or central fishes depending on the block. Auditory and visual feedback was provided in a cartoon fashion to sustain a high attentional level. Dependent variable: RT/percentage of hits in incongruent condition in flanker blocks.
	Local-Global [81]	This task was adapted from [82, 83]. Participants were instructed to respond to either global figures (block 1) or local figures (block 2) or to alternate their attention between global and local figures (block 3). The global figures consisted of large squares or triangles made up of smaller shapes (squares or triangles as local figures). In the congruent condition, the global and local shapes were the same (e.g., a large l triangle composed of small triangles), while in the incongruent condition, the global and local shapes differed (e.g., a large triangle composed of small squares). Participants responded by pressing a key corresponding to either the global or local shape, depending on the block. Dependent variable: RT/percentage of hits in incongruent conditions in local blocks.
	Receptive attention [84]	This task is a subscale of the Cognitive Assessment System (CAS). Seven-year-old children were presented with four sheets containing 200 pairs of drawings, which they completed under two different conditions. In the first condition, they were asked to underline the physically identical drawings, while in the second condition, they had to identify and underline drawings that belonged to the same lexical category. For children aged 11 and 15, the task involved 400 pairs of letters. In condition 1, they underlined the physically identical letters, and in condition 2, they selected the letters belonging to the same lexical category. Dependent variable: base score (number of correct answers minus the number of mistakes and the time to complete the test.
Prepotent response inhibition	Go no-go	This task was adapted from [85]. Children were instructed to press a key when presented with go stimuli (t-shirts representing the national football teams of Peru, Argentina, Brazil, Germany, France, and the Netherlands and to withhold their response for no-go stimuli (the t-shirt of Spain’s national football team). Feedback on false alarms, inattentive responses, and anticipatory errors was provided after each trial. Dependent variable: percentage of hits to no-go trials.
	Stop_Signal [86]	The STOP_IT software [87] was utilized. Participants were tasked with distinguishing between a square and a diamond in two different conditions. In non-signal trials, they had to press the key corresponding to the presented shape. In the stop-signal condition, the lines of the shapes thickened after a variable delay, and participants were required to refrain from responding. On stop-signal trials, a stop signal was presented after a variable SSD (stop-signal delay). SSD was initially set at 250 ms and was adjusted continuously with the staircase tracking procedure: when inhibition was successful, SSD increased by 50 ms; when inhibition was unsuccessful, SSD decreased by 50 ms. Dependent variable: stop-signal reaction time (SSRT).
	Stroop [88]	A computerized Stroop task using two colors was designed according to [89]. Participants were asked to name the ink color and press the corresponding key. In the neutral conditions, participants are asked to name the ink color of an array of five asteriscs printed in blue or red. In the congruent condition, the ink color (blue or red) matched the word printed (e.g., the word “red” printed in red color). In the incongruent condition, the ink color differed from the word (e.g., the word “red” printed in blue color). Dependent variable: RT/percentage of hits in the incongruent condition.
Cognitive inhibition	Updating information in working memory task (intrusions) [90]	We used a modified version of the task developed by [57]. Participants listened to 24-word lists, each containing 12 words, presented at a pace of one word per second. Each list included words to be recalled, discarded, and filler words. This task allows to differentiate between maintenance and inhibition components of the updating process by varying demands on memory load and suppression. The number of words to be recalled differed by condition: three in the low-load condition and five in the high-load condition. Similarly, the number of words to be discarded varied between two in the low-suppression condition and five in the high-suppression condition. The number of filler words ranged from two to seven. Dependent variable: previous-list intrusions.
	Negative Priming	We utilized a modified version based on [60, 91]. Drawings of objects and animals, printed in red, green, or black, were arranged into prime and probe displays. Participants were instructed to ignore the red shape (distractor) and focus on responding to the green shape (target). Each experimental session included a pair of prime-probe trials. During these trials, two overlapping green and red shapes were shown alongside a comparison shape in black. Participants had to determine if the green shape matched the black one while ignoring the red shape. In the ignore condition, the stimulus to be ignored in the probe trial was the same as the one to be attended to in the prime trial. In the control condition, the stimulus to be ignored in the probe trial was different from the one to be attended to in the prime trial. Dependent variable: mean RT/percentage of hits in the ignored condition.
Processing speed	Stroop	We used the neutral condition of the Stroop task. Dependent variable: mean RT/percentage of hits
WMC	Reading Span Task [92]	We used an adapted version for children of the classical task [93]. Participants had to read aloud each sentence at their own pace and remember the last word of the sentence. The task consisted of 48 phrases (6 training and 42 experimental) grouped into levels of 2, 3, 4, and 5 sentences, with three series of sentences for each level. At the end of each series, participants had to remember the last word of each sentence in the same order as presented. The reading span is the highest level at which the participant can accurately remember at least 2 out of the 3 series. Dependent variable: reading span
	Counting Span Task [94]	We used an adapted version for children of the classical task [94]. Participants were shown 48 visual displays consisting of red and blue squares, organized into levels of 2, 3, 4, and 5 displays each. For each level, three series of displays were presented. Participants had to count the blue squares. At the end of each series, they had to recall the number of blue squares in each display at that level, in the order they were presented. The counting span is the highest level at which the participant can accurately remember at least 2 out of the 3 series. Dependent variable: counting span

*Note*. These tasks are part of a larger research program, with some being utilized in previous studies and detailed in earlier publications that aimed to address different research questions [57, 95–98].

### Procedure

Participants were tested individually in a quiet room during school hours across four different sessions. The task order was counterbalanced across participants. The stimuli were balanced in each task so that there was an equal number of answer types per condition; the order of stimuli within a task was also randomized (see Supplementary Materials. Section A). In all the tasks—except the receptive attention task—stimuli were computer-administered. Randomization and time were controlled through E-Prime software, version 2.0 (Psychology Software Tools, Inc., 1996–2002). Six trained examiners administered the tasks. Although all tasks included practice trials before the experimental ones, the examiners did not start the experimental block of trials without verifying that the children fully understood the task.

## Results

### Data preparation

For data trimming, we followed the same procedure as [99]. We carried out this procedure in several steps. (1) Only trials on which correct responses were given were analyzed for the RT measures, and RTs < 200 ms were eliminated. (2) Timing tasks (except stop signal, which did not depend on a mean RT) were analyzed using a trimming procedure that is robust to nonnormality to obtain the best measure of central tendency [100]. (3) Mean scores above three standard deviations (SDs) from the mean age group were replaced with values of the group mean plus three SDs. This affected 6.16% of the experimental scores (6.6%, 5.3%, and 6.6% for 7-, 11-, and 15-year-olds, respectively) and 5.33% of the neutral scores (6%, 7.3%, and 2.66% for 7-, 11-, and 15-year-olds, respectively). After these transformations and trimmings, the variables showed acceptable skewness and kurtosis (see a complete descriptive analysis in Supplementary Materials section B). All measures were converted to z-scores. Further, to account for the speed-accuracy interactions, we used the Inverse Efficiency Index, in which the RTs are divided by the percentage of hits, both in experimental and neutral conditions [101]. The Inverse Efficiency Index is an integrative score that improves control for speed-accuracy trade-offs, making it particularly suitable for samples involving children and older adults [101]. Although the Inverse Efficiency Index has faced criticism from some researchers [102] more recent studies have affirmed its validity with error rates below .10 [103, 104]. In our study, error rates were below this threshold [105], for further discussion].

### Analytic strategy

#### Preliminary analysis

A set of preliminary analyses was completed to test the suitability of the tasks by checking their reliability computing by Cronbach’s alpha, and the ability to detect age-related changes through ANCOVAS. Descriptive and correlational analyses were also conducted (for specific details see Table 3 and S3 Table in S1 File). The task’s reliability was good across age groups (see precise estimates in Table 3). S3 Table in S1 File shows the proportions of correct answers and RTs for the inhibition tasks in congruent and incongruent conditions for each age group. ANCOVAs showed that in all tasks, congruent conditions were significantly easier than incongruent ones. They also showed a significant improvement in performance from 7-year-olds to 15-year-olds in the covariates processing speed and WMC. Concerning inhibitory tasks, the age-related pattern of single inhibition tasks was not uniform, showing subtle variations across tasks. Receptive attention, flanker, Stroop, and negative priming tasks showed continuous increments from 7-year-olds to 15-year-olds, revealing that performance in these tasks continues to improve across adolescence independently of processing speed and WMC. However, local-global, stop signal, go-no go, and intrusions in WM tasks showed increments only up to 11 years of age. S4-S6 Tables in S1 File show the correlations and partial correlations among the variables. Correlations follow the same pattern of low and moderate coefficients expected in executive functioning in agreement with previous studies [30, 71, 76, 77, 106]. Although lower, most partial correlations among inhibitory tasks remained significant after controlling processing speed and WMC.

**Table 3:** Descriptives and task’s reliability (Cronbach’s α).

			7 year-olds					11 year-olds					15 year-olds				
Construct	Task (dependent variable)	measure	Mean	Standard deviation	Skewness	Kurtosis	Reliability	Mean	Standard deviation	Skewness	Kurtosis	Reliability	Mean	Standard deviation	Skewness	Kurtosis	Reliability
**resistance to distractor interference**	**Flanker** (incongruent flanker RT/ACC)	RT	1276	346	.56	.86	.98	714	220	.97	1.03	.99	488	104	1.44	2.22	.99
		Accuracy	.90	.09	-1.09	1.41	.86	.931	.07	-1.38	2.16	.87	.969	.05	-2.73	9.70	.8
	**Local-Global** (incongruent local RT/ACC)	RT	1033	296	1.05	.64	.98	655	141	.92	.94	.99	531	86.3	.82	.75	.98
		Accuracy	.91	.08	-.96	.56	.89	.931	.07	-1.38	1.85	.89	.954	.06	-2.05	5.8	.77
	**Receptive attention** (Base Score)		34.1	7.38	.17	.03	.63	3.9	9.8	. 70	1.65	.88	48.7	11.5	.13	-.18	.79
**Response inhibition**	**Go no-go** (Accuracy go)		.79	.11	-.39	-.50	.89	.87	.1	-1.29	2.41	.81	.90	.07	-1.8	4.64	.74
	**Stroop** (incongruent RT/ACC)	RT	1198	372	.74	.20	.86	766	210	.87	.45	.95	615	16	1.01	.80	.96
		Accuracy	.97	.05	-2.48	7.6	.66	.986	.03	-3.27	13.9	.64	.991	.02	-3.52	16.2	.41
	**Stop signal** (SSRT)	SSRT	516	233	.21	-1.25	.91	324	141	1.31	2.19	.93	332	158	.62	-.07	.98
**cognitive inhibition**	**Negative priming** (ignored RT/ACC)	RT	1497	266	.75	1	.94	1069	229	.64	.538	.97	832	152	.39	-.32	.98
		Accuracy	.90	.06	-1.26	1.97	.81	.910	.06	-.50	-.160	.77	.940	.04	-.97	1.01	.66
	**Updating WM task** (Intrusions errors)		.08	.08	1.33	1.47	.89	.05	.05	1.95	4.97	.91	.02	.03	1.37	1.38	.87
**Processing speed**	**Stroop** (neutral RT)	RT	864	211	1.02	1	.87	610	133	1.08	1.65	.8	517	102	.52	-.29	.9
**WMC**	**Reading span task** (Reading span)		2.57	.37	-.05	-.71	.42	2.76	.42	-.01	-.47	.57	3.21	.51	-.17	-.54	.52
	**Counting span Task** (Counting span)		3.64	.63	.18	-.04	.58	3.85	.60	.03	-.02	.73	4.61	.60	-.86	-.30	.63

*Note*. We computed the Inverse Efficiency Index (RT/ACC) as the dependent variable for speeded tasks (flanker, local-global, Stroop, and negative priming). The slowest RTs show lower performance. The dependent variable for updating is errors. The dependent variable for go no- go, reading, and counting span tasks is accuracy. Reliability was computed by Cronbach’s alpha.

#### Multi-group confirmatory factor analysis to test age-related changes in the organization of inhibitory functions

As data were not normally distributed, we used maximum likelihood estimation with robust (Huber-White) standard errors. Goodness-of-fit of each model to the data was evaluated via global model fit indices that adjust for nonnormality: the Yuan-Bentler correction factor for the chi-square statistics (YB χ2), the robust comparative fit index (the robust CFI; [107] and the robust root mean square error approximation (the robust RMSEA; [107] and its 90% confidence interval (90% CI). The fixed variance method of identification was used in all models [108].

The Yuan-Bentler correction was used to test the exact-fit hypothesis and prove that there is no difference between the model-implied covariance matrix and the population covariance matrix. A non-significant p-value (p ≥.05) supports the exact-fit hypothesis. The robust RMSEA is an absolute fit index where a zero value supports the exact-fit hypothesis (values >.08 are considered a poor fit, values in the range of .05 to .08 are considered an adequate fit, and values ≤ .05 support the close-fit hypothesis). Ideally, the 90% CI for the robust RMSEA should not include values considered as poor fit [109]. The robust CFI assesses how the specified model improves the fit over the null model (values >.90 are considered an acceptable fit, while values >.95 are considered a good fit [110].

For all the models, we tested the configural invariance hypotheses across age groups. According to this hypothesis, both the number of factors and the correspondence between factors and the measured variables are the same across groups. Thus, all parameters are freely estimated (e.g., factor loadings, latent and observed means (intercepts), residual variances, covariances, etc.) in each group, except those used to identify the factor structure. The next step in testing measurement invariance (metric invariance) consists in examining a model with regression weights (i.e., factor loadings) being invariant across groups. In this respect, factor loadings significatively varied across age groups and therefore, this level of measurement invariance was not retained (data not shown) and it is not allowed to examine the next steps for measurement invariance.

To pursue our first and second research questions, a series of multigroup confirmatory factor analyses were conducted to test the factorial structure of inhibitory functions in the different age groups, first without controlling processing speed and WMC (first research question), and then controlling for these factors (second research question). Finally, to address our third research question, we contrasted the models to test whether imposing restrictions on processing speed and WMC makes a statistically significant difference in model fit, both within (Likelihood ratio test) and between ages (Akaike’s Information Criterion; AIC).

Multigroup confirmatory factor analyses were performed in Rstudio [111] using different R packages [112–114]. The fixed variance method of identification was used in all models [108]. We examined the significance and strength of parameter estimates wherein correlations and factor loadings between 0-±.20, ±.21- ±.40, ±.41-±.60, ±.61-.99, and 1 were considered weak, low, moderate, strong, and perfect, respectively. The goodness-of-fit criteria for Multigroup confirmatory factor analyses are detailed in Supplementary Materials. Section C.

**Fig 1:**
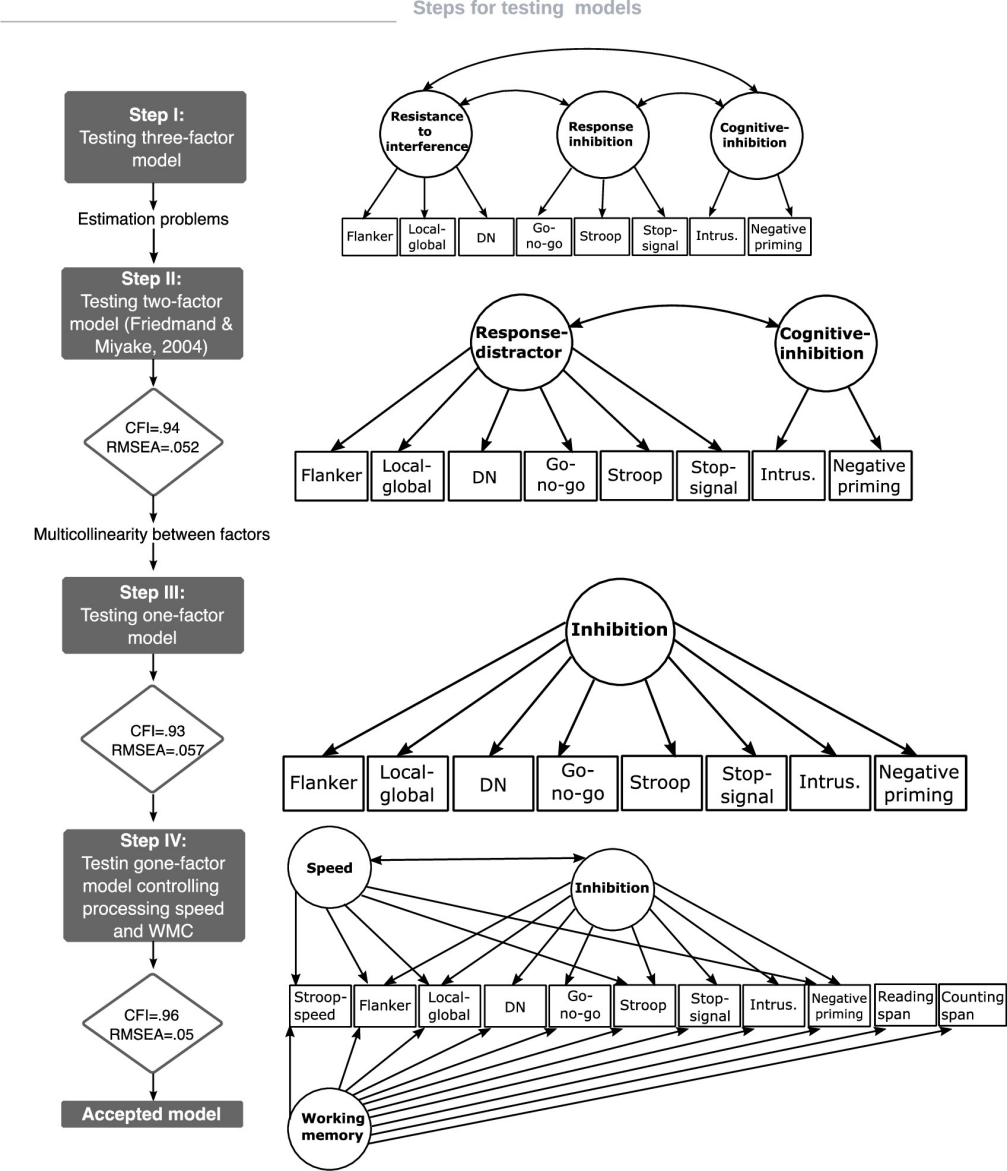
Model testing sequence. CFI: robust comparative fit index; RMSEA: robust root mean square error of approximation; Response distractor: response distractor factor; Cognitive inhibition: cognitive inhibition factor; Speed: processing speed factor; Inhibition: inhibition factor; WMC: WMC factor; Stroop neutral: response time for neutral condition of Stroop task; flanker: response time/accuracy for incompatible condition of flanker task; local-global: response time/accuracy for incompatible condition of local-global task; DN (receptive attention): accuracy; go-no-go: accuracy no go trials; Stroop: response time/accuracy for incompatible condition of the Stroop task; stop-signal: SSRT stop signal reaction time; Intrus.: number of words incorrectly recalled in the updating WM task; negative priming: response time/accuracy for the ignored condition of the negative priming task; Reading span: Reading Span for Reading span task; counting span: counting span for counting span task.

We tested the configural invariance hypotheses across age groups for all models, assuming that the number of factors and the correspondence between factors and the measured variables are the same across groups. As the factor loadings significatively varied across age groups, it is not allowed to examine the next steps for measurement invariance (see Supplementary Materials. Section C for a detailed description of the configural invariance hypotheses). Thus, assuming the configural invariance hypotheses do not allow formally comparing the variances, covariances, and means of factors across age groups. Fig 1 shows the model testing sequence for examining the factor structure of inhibition measures used as indicators. Steps I to III are related to research question 1 and Step IV with the research question 2.

As requested by one of the reviewers, we also fitted all the models using difference scores. Fit indices are presented in S9 Table in S1 File. However, these models did not show a good fit, likely due to psychometric issues commonly associated with difference scores, such as low between-participants variability, poor reliability, and high measurement error. These issues can result in low correlations and hinder the ability to identify a robust factor [105, 115, 116]. For interested readers R scripts for these models are available at: https://osf.io/7f25r/?view_only=ab4999740e364c67a7f7494968753141.

*Steps I and II*. Although we have pointed out some limitations of previous findings [67, 68], we tested a model with three correlated factors: resistance to interference (flanker, local-global, receptive attention), response inhibition (go-no-go, Stroop, stop signal), and cognitive inhibition (intrusions, negative priming). The covariance matrix of latent variables was not positive definite in the three age groups; therefore, there were no eigenvalues, and no solution was possible.

Our second candidate model was a two-factor model that resembled the final model proposed by [30] for young adults (see Fig 1, Step II). It included two correlated factors: response-distractor and cognitive inhibition. S10 Fig in S1 File shows factor loadings and correlations in each age group. The fit of the model to the data was acceptable (Yuan-Bentler correction factor χ^2^_(57)_ = 81.745, *p* = .018; robust CFI = .943; robust RMSEA = .055[.024 - .81]). The correlation between response distractor and cognitive inhibition was strong for the 7 and 15 age groups (*r* = .97 and *r* = .88, respectively). These strong correlations suggest that testing the one-factor hypothesis for our inhibition measures is reasonable. Thus, in Step III we tested a model in which a factor that we termed “*inhibition*” explained the variability of all inhibition measures.

*Step III*: *Fit a one-factor model*: *Inhibition*. S11 Fig in S1 File displays the configural invariance model for the one-factor model. The Yuan-Bentler correction factor χ^2^ statistic did not allow to retain the exact-fit hypothesis (Yuan-Bentler correction factor χ^2^(60) = 90.351, *p* = .007), and the robust CFI suggested an acceptable fit of the model to the data (robust CFI = .93). The robust RMSEA indicated an adequate fit, but the 90% CI included values for the poor fit hypothesis (robust RMSEA = .06 [.032-.084]).

*Step IV*: *Fit a one-factor model controlling for processing speed and WMC*. We examined the role of processing speed and WMC in inhibition measures.

Our initial objective was to employ the neutral blocks as a measure of processing speed associated with each conflict task (Stroop, flankers, local-global, and negative priming). To achieve this, we fit several models where the neutral condition of each speed-based inhibition task served as an indicator of the processing speed factor (see S14 and S15 Figs in S1 File). Although the fit of these models to the data was good, the strong correlations between some incongruent conditions and their corresponding neutral conditions hindered our ability to discern the underlying processes of these latent variables and made it difficult to rule out that the shared variance could be attributed to task-specific attributes common to both conditions in the flanker, local-global, and negative priming tasks, beyond processing speed alone [106, 117].

Consequently, to reduce task-specific variance and mitigate this potential confound, we decided to avoid using neutral/control conditions, opting instead for elementary tasks as measures of processing speed [47, 118, 119]. Specifically, we chose the condition with minimal executive demands, which in our case was the neutral condition of the Stroop task. This approach enhances comparability with other developmental studies on executive functioning that used the same measure as control of processing speed [54, 120].

Assuming that perceptual speed could primarily influence RT measures in inhibitory functioning tasks [50, 73], we used the neutral condition of the Stroop task to control processing speed in each RT task (flanker, local-global, Stroop, and negative priming). We did not control for processing speed in the DN task (as time has already been corrected in the base score), Stop Signal task (since SSRT is not influenced by reaction times), go no-go task (due to specific feedback allowing adaptive responses independent of speed, with correct responses recorded), and in the updating in working memory task (recall of word lists under no time pressure).

**Fig 2:**
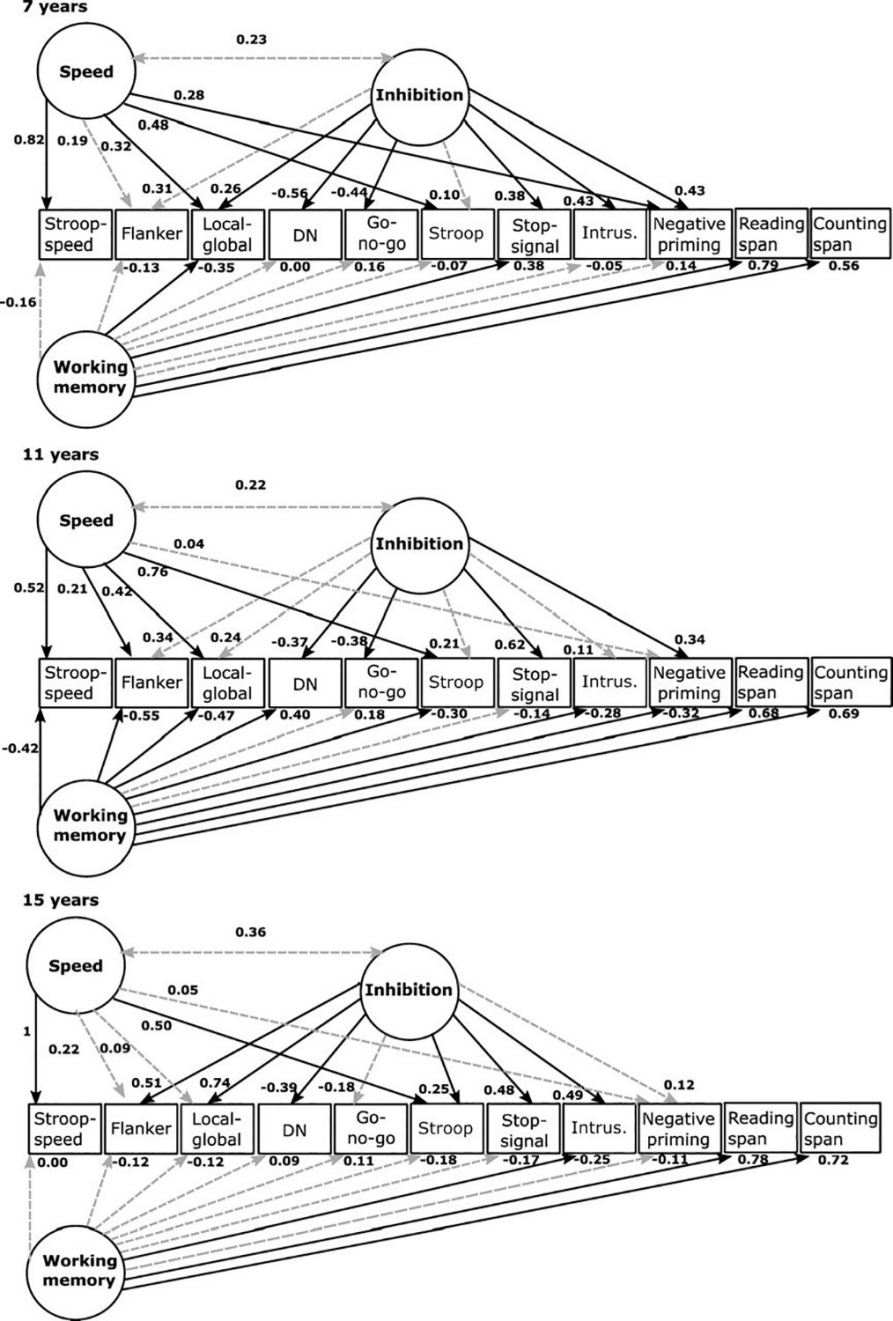
WMC-speed-inhibition model. Dashed-gray lines indicate non-significant factor loadings (p > 0.05). Speed: processing speed factor; inhibition: inhibition factor; Working memory: WMC factor: reading span task and counting span tasks.; speed factor: Stroop neutral: response time for neutral condition of Stroop task; inhibition factor: flanker: response time/accuracy for incompatible condition of flanker task; local-global: response time/accuracy for incompatible condition of local-global task; DN (receptive attention): accuracy; go no-go: accuracy no go trials; Stroop: response time/accuracy for incompatible condition of the Stroop task; stop-signal: SSRT stop signal reaction time; intrus.: number of words incorrectly recalled in the updating WM task; negative priming: response time/accuracy for the ignored condition of the negative priming task.

Fig 2 displays this configural invariance model. The variance of the Stroop-neutral condition for the 15-year age group was restricted to be > 0 because the initial model showed a negative variance estimate (a.k.a., Heywood case). To evaluate the possibility of structural misspecification, we looked at the CI from the robust maximum likelihood estimate of the negative error variance estimate. The CI included positive values suggesting that the population variance is positive but near zero and that the negative estimate can be the result of chance [121]. This finding can be interpreted as evidence of a correct structural specification. Thus, we assume that negative variance is not related to structural misspecification, and, therefore, we were confident about the model estimates. A priori Monte Carlo simulation analysis was conducted in SemTools [122] to evaluate the statistical power of a three-factor model. Two thousand random samples were generated under the following conditions: 1) sample size varied from 100–500 participants (5 replications for each sample size); three factors (correlations varied from 0.2 to 0.3); and factor loadings varied from 0.3 to 0.7. The simulation study indicated that a sample size of 300 participants was required to achieve a *RMSEA* = 0.042 and a *CFI* = 0.959.

The exact-fit hypothesis cannot be retained (Yuan-Bentler correction factor χ^2^_(90)_ = 126.57, *p* = .007). The robust CFI showed a good model fit to the data (robust CFI = .96). The robust RMSEA supported the close-fit hypothesis (robust RMSEA = .049[.027-.068]) while the 90% CI did not include values considered as a poor fit. The processing speed and WMC tasks were significant and showed moderate to strong factor loadings on their respective latent variables. The Stop-signal and receptive attention were the only two tasks that significatively loaded onto the inhibition factor across the three age groups. These tasks had low to strong factor loadings ranging from ±37 to ±62. For the 7-year group part of the variance of the stop-signal task was explained by working memory, whereas in the 11-years group, part of the variance related to the receptive attention task was due to working memory. In the 7-year group, the flanker and Stroop tasks did not significantly load on the inhibition factor while, in the 11-year group, four of eight measures did not significantly load on the inhibition factor (flanker, local-global, Stroop, and intrusions). In the 15-year group, the go no-go and negative priming tasks did not significantly load on the inhibition factor. There were no significant correlations among factors in the three age groups.

Following a reviewer’s suggestion, we fitted an alternative model that constrained the correlation between the factors to zero (WM-speed-Inhibition restricted model). The fit of this model to the data was good (YB χ^2^(93) = 135.13, p = .003; robust CFI = .953; robust RMSEA = .053 [.032 - .071], AIC = 9963.402, Delta AIC = 4.981). However, the Delta AIC suggests moderate evidence in favor of our final model presented in Fig 2, in which the correlations between the Speed and Inhibition factors were freed. Factor loadings for the WM-speed-Inhibition restricted model were similar to those in the final model. Factor loadings for the restricted WM-speed-Inhibition model were similar to those in the final model presented (WM-speed-Inhibition model). This model is presented in S13 Fig in S1 File.

Table 4 summarizes the goodness-of-fit of all tested models. As the table shows, the WMC-speed-inhibition model is the best account to the data. Therefore, we examine the role of speed and WMC on this model.

**Table 4:** Goodness-of-fit of the tested models.

Model	YB χ^2^	*df*	*p*	Robust CFI	Robust RMSEA
Two-factors model	81.745	57	.018	.943	.052[.016 - .79]
One-factor model	90.351	60	.007	.935	.057[.027-.083]
**WMC-speed-inhibition model**	**129.87**	**90**	**.004**	**.959**	**.05[.029-.069]**

Note. Robust CFI: the robust comparative fit index; df: degree of freedom; the robust RMSEA: the robust root means square error approximation; YB χ2: Yuan-Bentler correction factor for the χ2. The 90% confidence intervals of the robust RMSEA are given in brackets. The Three-factors models are not displayed due to previously described problems.

*Exploring the influence of the WMC and processing speed for solving inhibition task*. We examined the relative importance of processing speed and WMC across age groups for informing research question 3. Thus, we implemented a series of models in which the factor loadings of inhibition tasks on processing speed and WMC were, one at a time, constrained to zero in each age group (a schematic representation is given in S12 Fig in S1 File). Specifically, factor loadings of inhibition tasks on processing speed were independently forced to be zero in the 7-year-old group (model M1), the 11-year-old group (Model M2), and the 15-year-old group (model M3). In models M4, M5, and M6, factor loadings of inhibition tasks on WMC were independently fixed to zero for the 7-, 11, and 15-year-old groups, respectively. These models were used as input for calculating the differences between models ΔAIC and for the Likelihood Ratio Test (S7 Table in S1 File shows the Likelihood ratio test between the WMC-speed-inhibition model and each restricted model). AIC and the BIC differ in terms of penalty for model complexity. The latter criterion imposes a harsher penalty than the former and it is well-known that it favors less complex models. In this study both criteria selected the same model, and they did not differ to a large degree in terms ΔAIC and ΔBIC. For simplicity and the good properties, we present only the AIC values.

Table 5 shows the model testing sequence. The top section of Table 5 displays the AIC and ΔAIC of models in which the factor loadings of inhibition tasks on processing speed were restricted to zero. ΔAIC suggests that the best candidate in the set was M2 (i.e., 11-year-olds, ΔAIC = 0). Thus, relative to model M2, the ΔAIC provided less support for model M1 (ΔAIC = 3.918) and no support for model M3 (ΔAIC = 10.525). This, in turn, suggests that the role of processing speed in solving inhibition tasks is more relevant for children in the 15-year-old group than for children in the 7-year-old group. Therefore, the order of relevance of speed is from the 15-year-olds, 7-year-olds, and 11-year-olds group.

**Table 5:** Model testing sequence for examining the role of processing speed and WMC across age groups.

**Model name**	***YB* χ** ^**2**^	***df***	***p***	**Robust CFI**	**Robust RMSEA**	**AIC**	**ΔAIC**
Speed constrained to 0							
M1(7 years)	152.290	94	.000	.936	.062[.043-.079]	9976.7	3.918
**M2 (11 years)**	**153.409**	**94**	**.000**	**.937**	**.061[.043-.078]**	**9972.8**	**0**
M3 (15 years)	155.168	94	.000	.931	.064[.045-.082]	9983.3	10.525
WMC constrained to 0							
M4 (7 years)	161.762	98	.000	.930	.063[0.045–0.080]	9977.4	20.129
M5(11 years)	171.082	98	.000	.918	.068[0.051–0.085]	9989.0	31.801
**M6 (15 years)**	**125.327**	**95**	**.017**	**.964**	**.046[0.019–0.066]**	**9957.2**	**0**

*Note*. Robust CFI: the robust comparative fit index*; df*: degree of freedom; the robust RMSEA: the robust root means square error approximation; *YB* χ^2^: Yuan-Bentler correction factor for the χ^2^. The 90% confidence intervals of the robust RMSEA are given in brackets.

The bottom section of Table 5 displays the AIC and ΔAIC of a series of models in which the factor loadings of inhibition tasks on WMC were restricted to zero. ΔAIC revealed that model M6 was the best account of the data (ΔAIC = 0) and that models M4 (ΔAIC = 20.129) and M5 (ΔAIC = 31.801) did not receive support. These findings suggest that WMC could play a more critical role in solving inhibition tasks for children in the 7- and 11-year-old groups than those in the 15-year-old group.

## Discussion

This study aimed to explore the organization of inhibitory processes during school ages, in three age groups, 7–8, 11–12, and 14–16, when controlling for WMC and processing speed. However, despite its importance for cognitive development and complex cognition, no studies have previously studied the factor structure of the three constructs across school ages, which is an important contribution of the present study. Crucially, we have also addressed some methodological concerns about the research in executive functioning in general and inhibition in particular [21, 105]: a) we used the same tasks across ages; b) we avoided differential scores; c) we addressed the trade-off between speed and accuracy in RT tasks; and d) we reduced the demands of WM to help participants remain active, maintain and remember the rules of the task. Moreover, all the tasks used are reliable, sensitive to age-related differences, and timing tasks have internal consistency as they showed reliable differences between compatible and incongruent conditions.

### What is the factor structure of the inhibition-related processes, and how does it progress from 7 to 15 years?

Concerning age-related differences in inhibitory abilities, ANCOVA’s results corroborated previous studies that reported an increment of the different inhibitory abilities during school ages [20, 27, 45, 49, 53, 71]. Regarding the structure and organization of inhibitory abilities, confirmatory factor analysis results indicated the suitability of a configural invariance model with a common single factor for inhibitory functions, which is invariant across ages. Thus, our findings suggest some commonality between the three postulated inhibitory constructs, as general inhibition [123] and the executive attention view of WMC [14, 15, 124, 125] have been proposed.

Therefore, our results corroborated neither the early dissociation between response inhibition and resistance to distractor interference found by [67, 68] nor the dissociation between response-distractor and cognitive inhibition found by [69] in children and [30] in young adults.

One possible explanation for these discrepant results could have to do with methodological issues as different authors use divergent criteria to retain two highly correlated factors as separate or collapse them into one factor [30, 67]. Also, in different studies, the same task is used to load different constructs (e.g., Tiego et al. [71] used the Stroop task as an indicator of resistance to distractor interference, whereas Gandolfi et al. [67] used a Stroop-like task as an indicator of response inhibition]. Some studies also included as an inhibition indicator some tasks that are mainly devoted to measuring another construct, as it seems to be the case of the dimensional change card sort used by [67] mainly devoted to measuring task switching [31]. Still, the dissociation could be attributed to an artifact in the measurement modality as it could occur in the study of [30] where response-distractor indicators are all speeding tasks and cognitive inhibition indicators are all accuracy tasks, and consequently, the dissociation could be attributed to differences in processing speed and not differences in “pure” inhibitory control.

Another possible explanation is more theoretically grounded. The lack of differentiation may indicate that various inhibitory abilities depend on active goal maintenance, and thus on WMC or processing speed, particularly during the developmental course [33, 43, 44, 50, 53, 71].

Thus, our results did not support a progressive age differentiation in the structure of inhibitory functions until the age of 15–16, as postulated in H1 based on different previous studies [6–8, 31] and on the strong version of the differentiation hypothesis [75] that refers to changes in the structure of the factors and relationships among factors. However, our results supported a weak version of the differentiation hypothesis [77] referred to changes in the strength of correlations between factors and variations in factorial loadings across ages in agreement with other previous studies where no strong evidence of differentiation was found [126].

### What is the factor structure of the inhibition-related processes, and how does it progress from 7 to 15 years when controlling for WMC and processing speed?

A new model was tested to address this research question (Step IV), in which inhibition is controlled both by processing speed and WMC. We did not find evidence for differentiation among inhibitory functions when controlling WMC and processing speed, but this does not mean that inhibition, processing speed, and WMC are the same constructs. Instead, our results showed that the three processes interact to achieve adaptive behavior [2, 3]. Importantly, however, our results support the partial dependence of inhibitory control on processing speed and WMC and that processing speed and WMC contribution to resolving inhibitory tasks varied across ages (H3).

Our results contrast with the findings of [71], in which the differentiation between resistance to distractor interference and response inhibition at the age of 11 is partially dependent on a high-order WMC factor. Moreover, we think that subtle methodological differences could explain the discrepancy concerning our results. For example, Tiego et al. [71] used the Stroop task as an indicator of resistance to distractor interference, whereas we used it ―as most previous studies did― as an indicator of response inhibition [127]. Tiego et al. [71] also used different indicators for distractor interference (SS-conflict) and response inhibition (SR-conflict) of the same tasks (e.g., flanker task, which could also facilitate the dissociation between factors, an important issue to be addressed in future research.

### Does the contribution of WMC and processing speed in inhibitory tasks performance vary across ages?

To test the relative importance of WMC and processing speed across age groups, a new series of models were computed in which the factor loadings of inhibition tasks on processing speed and WMC were, one at a time, restricted to zero in each age group.

The comparison of successive models constraining to zero processing speed showed that, although processing speed contributed to performing the inhibitory task in the three age groups, it was more relevant for children in the 15-year-old group followed by children in the 7-year-old group. However, the contribution to processing speed could differ between these two groups. In one extreme, the slow processing speed of 7-year-olds could decrease processing efficiency, overwhelming WMC and making it difficult to inhibit, according to H3 and previous findings [2, 31, 72]. On the other extreme, 15-year-olds efficiency in most tasks could be attributed to their faster processing speed. In this case, higher speed efficiency would allow efficient WM [2], and the increasing WMC could also decrease the probability of making inhibitory errors [3, 31].

Our results contrasted those studies that maintain that processing speed mainly explains changes in inhibition [20, 49], but agreed with those that revealed its important contribution for solving inhibition tasks [53, 54]. Therefore, given that processing speed did not fully account for age-related changes in inhibition, one possibility is that general processing speed influences inhibition indirectly by increasing or decreasing WMC, an issue that deserves attention in future research.

On its part, the comparison of successive models constraining to zero WMC showed that WMC played a critical role in solving inhibition tasks for the 7- and 11-year-old groups, not in the 15-year-old group. We found that the 7-year-old group underperformed in the WMC tasks and that the WMC factor only loaded significantly on local-global and stop signal tasks. Thus, based on these findings, we can assume that owing to limitations in WMC, the inhibition tasks could have been more demanding for the 7-year-old group, which would confirm previous findings demonstrating that WM load directly decreases response inhibition [128]. Moreover, as increasing the WM load also decreases processing speed [129, 130], we conjecture that overwhelmed WMC could also indirectly influence the performance of inhibition tasks by increasing response times. Thus, in the case of the 15-year-old group, their increasing WMC and higher processing efficiency would decrease WM load in resolving inhibitory tasks, which decreases the probability of committing inhibitory errors.

This differential contribution of WMC for performing inhibitory tasks counters that of [70], who found that the factor loadings of WMC were similar across ages. A possible explanation of these discrepant results could be that these researchers used WM tasks that tap only maintenance, not manipulating information, which is a key component of WM that could contribute more to age-related differences.

Globally, our results showed that inhibitory control partially depends on general processing speed and WMC, but their relative contribution varied across ages, confirming H3. Importantly, it also showed that the interplay of processing speed, WMC, and inhibition in resolving inhibitory tasks could also be a reflection of the strategies used by children of different ages [53] based on the availability and use of attentional resources at that specific age [44, 45, 54]. This could be observed by closely examining the factorial loadings of the different tasks in the latent factors (Fig 2). As an example, consider the performance in the local-global task. ANCOVA results did not show significant differences between 11- and 15-year-olds. However, to get a similar good achievement in this task, 11-year-olds seem to rely mostly on WMC, whereas 15-year-olds seem to rely mostly on inhibition. Moreover, apart from the fact that children of different ages could be using different strategies to confront inhibitory tasks, our results could also be an indication that processing speed, inhibition, and WMC, although mainly independent processes, could share attentional resources, as is pointed out by the executive attention view of WMC [10, 14–16, 39–41].

## Conclusions

Across age groups, inhibition can be organized as a one-dimension factor in which processing speed and WMC directly modulate the variability of some inhibition tasks. This modulation changes across tasks could reflect the strategic use of attentional resources among the three processes. The partial reliance of inhibitory processes on processing speed and WMC demonstrated that the inhibition factor partially explains the variance of inhibitory tasks even when WMC and processing speed are controlled, and some methodological concerns are addressed.

## Limitations

Some researchers have pointed out that RTs are impure measures of cognitive processes and that their correlations could reflect multiple influences, not all due to the intended processes [50, 51]. Thus, they argued that it is unclear whether individual differences detected could be attributed to attentional control or differences in general processing speed or speed-accuracy trade-offs [50, 124]. Besides, RT differential scores have also been questioned due to their lack of reliability. In this study, we have tried to circumvent these problems by controlling for processing speed, using the Inverse Efficiency Index to account for the speed-accuracy interactions, and avoiding differential scores.

However, the decision to use the Inhibition Inverse Index of incongruent trials in four of the eight tasks employed in this study (flanker, local-global, Stroop, and negative priming) could be controversial for several reasons. One of the main concerns is that the interpretation of incongruent scores is complicated by confounds arising from variance unrelated to inhibitory processes, such as information processing, working memory demands, response caution, or the duration of perceptual and motor processes [50, 115]. These factors raise potential concerns about task purity and the validity of the construct [51].

Nevertheless, we have attempted to address these possible confounds by testing the models while controlling WMC and processing speed. Thus, we believe we can be quite confident that the construct represented by the eight indicators can be referred to as inhibition, especially considering that all eight inhibitory tasks demonstrate significant correlations despite differences in: a) Measurement methods: accuracy (receptive attention, go no-go), errors (intrusions) reaction time and accuracy (flanker, local-global, negative priming, Stroop), and stop-signal response time (Stop Signal); b) Stimulus modality: visuospatial (flanker, local-global, negative priming, stop signal, go no-go, receptive attention for 7-year-olds), visuospatial and verbal (Stroop, receptive attention for 11- and 15-year-olds), and verbal (intrusions in working memory); c) Types and sources of interference or distraction: perceptual (flanker, local-global, receptive attention), self-generated information (negative priming, intrusions in working memory), and habitual responses (Stroop, go no-go, stop signal); d) modality of response: key press (flanker, local-global, negative priming, stop signal, go no-go); oral responses (intrusions, reading span task, counting span, and Stroop); pointing at the screen (counting span), underscoring a drawing (receptive attention).

The series of multigroup confirmatory factor analyses confirm the consistency of these tasks. The robust fit of these models further supports their validity, aligning with our theoretical expectations. Consequently, it seems reasonable to hypothesize that these eight tasks, which are well-established measures of inhibitory-related processes [26, 27, 54, 60, 79, 80, 82–88, 90, 91, 131–140] collectively address a common cognitive construct: inhibition-related processes. What other shared cognitive processes could these tasks encompass, given their differences in measurement methods, stimulus modalities, sources of interference, and response modalities, as previously mentioned, and taking into account our control for WMC and processing speed?

However, given the current significant controversy regarding the measurement of attentional control, our results have to be replicated in future research using other different measurement models. As [15] pointed out, the reliable measuring of individual differences in attentional control presents a significant challenge for individual differences and developmental research. Thus, it is essential to refine the tools, tasks, and procedures we use, incorporating diverse statistical approaches to enhance measurement accuracy. Consequently, this issue should remain open for ongoing scientific investigation in the long run, rather than being definitively resolved in the short term [141].

The use of neutral trials in the Stroop task ensures that the speed factor derived from this task remains directly comparable to other RT tasks designed primarily to measure processing speed without additional cognitive load. This methodological approach promotes consistency in construct measurement across tasks, facilitating more robust and interpretable factor analyses. Additionally, it enhances comparability with other developmental studies on executive functioning that used the same measure as a control for processing speed [54, 120]. However, the common practice in confirmatory factor analysis is to use more than one measure as indicators, so relying solely on the neutral condition of the Stroop task as an indicator of processing speed could be a potential limitation in our study, which should be also addressed in future research.

Another possible limitation of the present study is the use of a cross-sectional design to explore age-related differences comparing children classified in groups based on age, as most of the studies in the field did. As some researchers claimed [70, 142], the classification in age groups could be masking individual differences and undermining differential performance among studies, a critical issue that also deserves more attention in future developmental research. Although this limitation did not invalidate the present findings, they need to be replicated, and longitudinal designs are needed to fully explore developmental differences.

Finally, we hope future studies systematically include processing speed and WMC as critical factors for understanding inhibition development, which would allow for a necessary replication of these results and clarify the interplay of the three processes in cognitive development.

## Supporting information

S1 File Contains all the supporting tables and supporting figures. (DOCX)
