# Supplementary material for: Age-related change in inhibitory processes when controlling working memory capacity and processing speed: A confirmatory factor analysis
Source: PLoS One. 2025 Jan 27;20(1):e0316347. doi: 10.1371/journal.pone.0316347 (PMC11771910; doi:10.1371/journal.pone.0316347)
Supplement: S1 File — (DOCX) [file pone.0316347.s001.docx]

**Table of Contents**

[Section A. Tasks 2](#_Toc181096872)

[1. Inhibition 2](#_Toc181096873)

[1.1. Resistance to distractors interference 2](#_Toc181096874)

[Flanker task 2](#_Toc181096875)

[Local-global Task 4](#_Toc181096876)

[Receptive attention task 6](#_Toc181096877)

[1.2. Response inhibition 8](#_Toc181096878)

[Go-no go task 8](#_Toc181096879)

[Stop-signal Task 9](#_Toc181096880)

[Stroop task 10](#_Toc181096881)

[1.3. Cognitive inhibition 11](#_Toc181096882)

[Updating information in Working Memory Task 11](#_Toc181096883)

[Negative Priming 12](#_Toc181096884)

[2. Processing Speed 14](#_Toc181096885)

[3. Working Memory Capacity 14](#_Toc181096886)

[Reading Span Test for children (PAL-N). 14](#_Toc181096887)

[Counting Span Task (CST) 16](#_Toc181096889)

[Section B. Preliminary analysis 17](#_Toc181096891)

[Descriptive Statistics 17](#_Toc181096892)

[Correlational analysis 21](#_Toc181096896)

[AN(C)OVA Analysis 22](#_Toc181096900)

[Section C. Multi-Group Confirmatory Factor Analysis (main analysis) 26](#_Toc181096906)

[Likelihood Ratio Test for Exploring the Influence of the WMC and Processing Speed for Solving Inhibition Tasks. 30](#_Toc181096911)

[Section D. Multi-Group Confirmatory Factor Analysis with neutral conditions as measure of processing speed 32](#_Toc181096913)

[Section E. Multi-Group Confirmatory Factor analysis with difference scores 36](#_Toc181096919)

[References 38](#_Toc181096921)

# Section A. Tasks

The tasks depicted are part of a larger research program, with some being utilized in previous studies and detailed in earlier publications that aimed to address different research questions (Carriedo et al., 2024; Carriedo, Corral, Montoro, Herrero, & Rucián, 2016; Carriedo, Corral, Montoro, Herrero, Ballestrino, et al., 2016; Herrero & Carriedo, 2018, 2019; Iglesias-Sarmiento et al., 2023).

1. **Inhibition**
   1. **Resistance to distractors interference**

**Flanker task**

Initially introduced by Eriksen and Eriksen (1974), several previous studies have used the flanker paradigm to obtain measures of response-distractor inhibition with children and adolescents (Huizinga et al., 2006; Rueda et al., 2004; Simonds et al., 2007; Waszak et al., 2010). The present version of the task is an adaptation from Munro et al. (2006) and Rueda et al. (2004). The stimuli consisted of five cartoon fish pointing to the right or the left, subtending a visual angle of 8º x 1.2º, horizontal and vertical, respectively. The color of the fish was blue or pink, depending on whether participants attended to the central fish or the flanker fish. A typical flanker task was administered in the central condition (blue fish), and the participants were to respond based on whether the central fish was pointing to the left or right, trying to ignore flanker fish at the same time, by pressing the corresponding left or right key on the keyboard ('Z' and 'M,' respectively) with their index fingers of both hands. A reverse flanker task was performed in the flanker condition (pink fish), in which the participants responded to the flanker fish' direction while ignoring the central fish.

The target fish was flanked by two fish noise stimuli on each side: (a) on congruent trials, the flanking fish were pointing in the same direction as the central one; (b) on incongruent trials, the flankers pointed in the opposite direction from the central fish; and (c) on neutral trials, the fishes to-be-ignored were replaced with geometrical figures without left-right defined direction (see S1 Fig). Each trial started with a cross-shaped fixation point; 500 ms later, an array of five fish appeared on the screen and remained until the participant responded. After the end of the trial, a pause of 500 ms ensued before the start of the next trial. The tasks consist of three phases: attention to the central fish, attention to the flanker fish, and alternating attention between the central and flanker fish. In the central attention condition, fishes are printed in blue color. The task started with a neutral central block (32 trials; the first 8 were warm-up trials not included in the analysis), continued with a practice block (12 trials) and two experimental blocks (24 trials each) in which participants had to respond to central fish. In the flanker phase, the color of the fish changed to pink, and the participants were instructed to respond to the flanker fish instead of the central fish. Similarly to the central condition, a neutral block (32 trials, first 8 were warm-up trials), a practice block (12 trials), and two experimental blocks (24 each) were successively administered. Finally, in the alternating phase, both central (blue) and flanker (pink) conditions were combined in the same blocks, with the fish's color cueing each trial's target stimuli. A practice block (24 trials) and two experimental blocks (48 trials each) were applied under this alternating condition. Auditory and visual feedback was provided in a cartoon fashion to sustain a high attentional level. Participants were instructed to respond as quickly as possible while making as few errors as possible. The dependent variable was the mean RT/percentage of hits in the experimental flanker block's incongruent condition. The average duration was 20 minutes.

**S1 Fig. Example of experimental and neutral conditions in the Flanker task.**

| 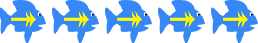 | Experimental Center Congruent |
| --- | --- |
| 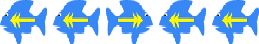 | Experimental Center Incongruent |
| 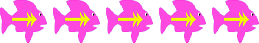 | Experimental Flanker Congruent |
| 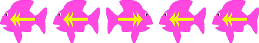 | Experimental Flanker Incongruent |
| 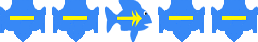 | Neutral for Center condition |
| 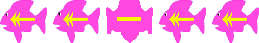 | Neutral for Flanker condition |

**Local-global Task**

Initially developed by Navon, (1977), two previous developmental studies (Huizinga et al., 2006; Mondloch et al., 2003) administered a local-global paradigm to children and adolescents to measure response-distractor inhibition. The present version of the task was adapted from (Mondloch et al., 2003; Montoro et al., 2011). The participants had to indicate the identity of the stimulus at the level to be attended (global or local), by pressing a keyboard button ('Z' for the triangle; 'M' for square) using their index fingers of both hands (see S2 Fig), in three different conditions (a) on congruent trials, the global and the local figures matched (e.g., global squares composed by local small squares); (b) in incongruent conditions shapes of global and local figures were different (e.g., global square composed by local small triangles) (c) in neutral trials, the fishes to-be-ignored were replaced with geometrical figures without left-right defined direction (see S2 Fig). At the bottom of the screen, a small triangle and a small square were displayed to remind this key assignment. The stimuli consisted of large squares and triangles (or diamonds, in the neutral local condition) made up of small squares and triangles (or diamonds, in the neutral global condition). The global stimuli subtended a visual angle of 2.7º x 2.7º. The global squares were made up of 24 local elements, the global triangles were composed of 18 local elements, and the global diamonds consisted of 16 local elements. The local elements subtended a visual angle of 0.3º x 0.3º and consisted of solid black figures on white background. Eight different stimuli were presented in this experiment. The experimental procedure consisted of three phases. In the first phase, participants responded to the global figure; in the second phase, they responded to the local elements. In the third phase, participants alternately responded to the global figure or the local elements. Each trial started with a cross-shaped fixation; 500 ms later, a visual stimulus was presented at the center of the screen until responses. Visual feedback was provided for every trial. The global phase started with a neutral block (32 trials; the first 8 were warm-up trials) that displayed large squares and triangles made up of smaller diamonds. After that, a global block practice (12 trials) and two experimental global blocks (24 trials) were administered. Similarly, the local phase started with a neutral block (32 trials; the first 8 were warm-up trials) that displayed large diamonds made up of smaller triangles or squares. Next, a local block practice (12 trials) and two experimental local blocks (24 trials) were administered. In the third phase, the participants performed an alternating global-local task that consisted of a practice block (24 trials) and two experimental blocks (48 trials), following an auditory cue presented before the stimuli. The spoken words 'BIG' or 'SMALL' indicated which level (global or local) the participants should respond to in each trial. Participants were instructed to make their responses as quickly as possible while making as few errors as possible. The dependent variable was RT/percentage of hits for incongruent conditions in experimental local blocks. The average task duration was 20 minutes.

**S2 Fig. Example of stimuli in Local-Global Task.**

| 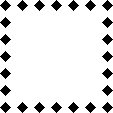  Neutral Square Global | 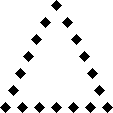  Neutral Triangle Global |
| --- | --- |
| 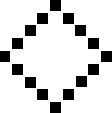  Neutral Square Local | 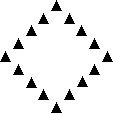  Neutral Triangle local |
| 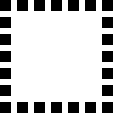  Experimental congruent | 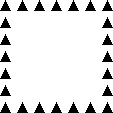  Experimental incongruent |
| 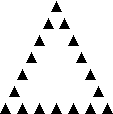  Experimental congruent | 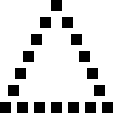  Experimental incongruent |

**Receptive attention task**


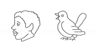

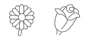
It is a scale of Cognitive Assessment System (CAS) (Naglieri & Das, 1997). This timed pencil-and-paper test measures the ability of selective attention requiring children to focus on relevant stimuli while ignoring irrelevant ones. 7-year-olds children were presented to 200 pairs of drawings per condition in four separate sheets comprising 50 pairs each. In the first condition, children needed to underline the physically identical drawings (e.g., they have to underline the drawing
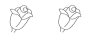
 , but not the drawing ). For the second condition, they had to underline the drawings that belong to the same lexical category (that have the same name) (e.g., they have to underline the drawing
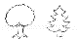
 , but not the drawing ) (see S3 Fig. Panel A). For 11 and 15-year-olds, a total of 400 pairs of letters per condition –physical identity and lexical identity– were presented in two separate sheets with 200 pairs each. For the first condition, participants needed to underline the physically identical pairs of letters (e.g., they had to underline *AA*, *aa,* but not *Aa*). For the second condition, participants had to underline the lexically similar pairs (e.g., they had to underline *Aa, AA, aa, but not Tr or tB*) (see S3 Fig, panel B). The task was individually handed out following manual administration, also providing scoring rules for each group of age. The dependent variable was the number of correct answers minus the number of mistakes and the time to complete the test, a *base score* that accounts for both correctness and speed. The task duration was 8 minutes.

**S3 Fig. Examples of Naglieri and Das stimuli for 7-year-olds (Panel A) and 11 and 15-year-olds (Panel B).**

Panel A


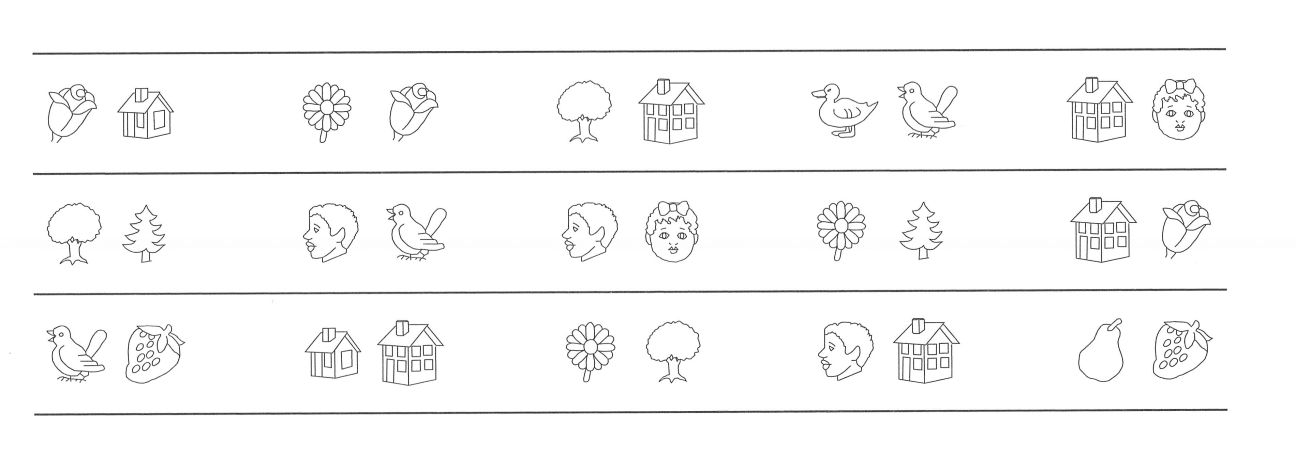


Panel B

| **tB** |  | **TT** |  | **Rb** |  | **ee** |  | **bN** |  |
| --- | --- | --- | --- | --- | --- | --- | --- | --- | --- |
| **RR** |  | **nE** |  | **nn** |  | **Tr** |  | **bt** |  |
| **EA** |  | **BB** |  | **TR** |  | **nb** |  | **aa** |  |

- 1. **Response inhibition**

**Go-no go task**

Three previous studies have used Go-no go tasks with samples of children and adolescents to measure response-distractor inhibition (Christ et al., 2006; Durston et al., 2002; Johnstone et al., 2005). The current version of the paradigm is an adaptation from (Christ et al., 2006). Two experimental conditions were provided: go and no-go. The no-go stimulus was the red t-shirt of the Spanish national football team, and the go stimuli were six t-shirts of other national football teams (i.e., Germany, Argentina, Brazil, France, Netherlands, and Peru) subtending approximately 4.3º horizontally and 5.3º vertically. On each trial, one of the t-shirts was centrally displayed. Participants were asked to press the space bar as quickly as possible when any stimulus except for the Spanish t-shirt appeared; in that case, participants had to avoid responding (see S4 Fig). After an interval of 1,000 ms, a new trial was presented. If a participant responded less than 100 ms after the presentation of a target (an anticipatory error), a visual message ("too fast, you cannot see the t-shirt yet") was displayed on the screen. In contrast, if a participant failed to respond within 1,500 ms (an inattentive error), a different visual message ("too slow, respond faster") was shown. If a participant responded on a no-go trial (a false alarm error), another visual message ("no response needed when you see the Spanish t-shirt") appeared. Following 49 go trials (neutral phase), six experimental blocks consisting of 40 trials (30 go and ten no-go trials) were administered. No-go stimuli was randomly included in 25% of the trials. At the end of each block, a break was offered. The dependent variable was the proportion of errors in no-go trials. Task’s duration was about 15 minutes.

**S4 Fig. An example of stimuli in the Go-no go task.**

| No go | Go |
| --- | --- |
| 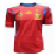 | 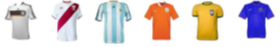 |

**Stop-signal Task**

Initially designed by (Logan & Cowan, 1984) three previous developmental studies have included stop-signal tasks as an index of response-distractor inhibition (Huizinga et al., 2006; St Clair-Thompson & Gathercole, 2006; Van Den Wildenberg & Van Der Molen, 2004). We used the software called STOP-ITa, developed by (Verbruggen et al., 2008), to run the stop-signal paradigm. The task consisted of no-signal trials (75% of the trials), in which participants had to discriminate between a square and a diamond. Participants were instructed to respond as fast and accurately as possible. The rest of the trials (25%) were stop-signal trials in which the lines of the shapes would become thicker after a variable delay, and subjects were instructed to withhold their responses (see S5 Fig). Each trial started with the presentation of the fixation sign, which is replaced by circles or diamonds after 250 ms. Participants had to press the "Z" key for *square* and "M" for *diamonds* shapes. The stimulus remained on the screen for 1,250 ms. The interstimulus interval was 2,000 ms. On stop-signal trials, a stop signal was presented after a variable SSD (stop-signal delay). SSD was initially set at 250 ms and was adjusted continuously with the staircase tracking procedure: when inhibition was successful, SSD increased by 50 ms; when inhibition was unsuccessful, SSD decreased by 50 ms. The experiment consisted of two phases: a practice phase of 32 trials and an experimental phase of three blocks of 64 trials. Between blocks, subjects needed to wait for 10 s before they could start the next block. The dependent variable was the stop-signal reaction time (SSRT). The task’s duration was about 15 minutes.

**S5 Fig. Example of stimulus in Stop-signal Task.**

| No signal | Stop signal |
| --- | --- |
| 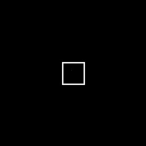 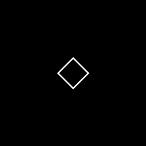 | 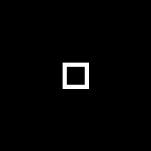 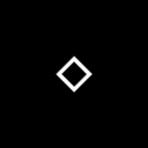 |

**Stroop task**

Designed initially by Stroop (1935), at least four previous works with children and adolescents have made use of the Stroop effect as a measure of response-distractor inhibition (Huizinga et al., 2006; Leon-Carrion et al., 2004; Rueda et al., 2005; Tamnes et al., 2010). We designed this version of the task following the recommendations provided by MacLeod (2006). Stimuli consisted of color items —asterisks (*****) and words "*red*" and "*blue*"— printed in blue and red colors in arial font and point size 32. Participants were then instructed to respond "red" or "blue" both vocally and by pressing "Z" and "M" keys with their left and right index finger as quick as possible. At the bottom of the screen, the letters "R" and "B" in gray color were displayed as reminders of this key assignment. First, a blank screen was presented for each trial lasting 500 ms. During this time, fixation point was shownfor the first 250 ms, . followed then by another blank for the last 250 ms. Next, the color item appeared in the center of the screen in the lower case (in the case of words). Finally, the color item was removed with the participant's response. The experiment consisted of three blocks of trials. The first block consisted of 60 neutral trials in which color items were five asterisks printed in blue or red colors. Participants were asked to state aloud and press the key associated with the color in which the asterisks were printed. Neutral trials were followed by 16 practice trials and then by 48 experimental trials. Half of the trials were congruent color items—that is, stimuli in which the color name matched the ink color (e.g., the word *blue* printed in blue color)— and the other half were incongruent trials in which the color name did not match the ink color (e.g., the word *blue* printed in red color). Congruent and incongruent conditions were intermingled randomly. The dependent variable was RT/percentage of hits in incongruent conditions. The average task duration was 10 minutes.

- 1. **Cognitive inhibition**

**Updating information in Working Memory Task**

We used an updating task originally devised by De Beni & Palladino (2004; Palladino et al., 2001), adapted for children and adolescents. The task provides some indexes of intrusions (previous-lists and same-list intrusions) that have been considered indexes of cognitive inhibition in previous developmental studies. Same-list intrusions have been considered an index of suppression of information in WM, and previous lists intrusions an index that accounted for proactive interference (Carriedo et al., 2016; De Beni & Palladino, 2004; Lechuga et al., 2006; Palladino et al., 2001, among others). The task contained 24 lists (20 experimental and four practice lists) of 12 words each. Each list included words to be recalled (relevant words), words to be discarded (irrelevant words), and filler words. The number of each kind of words in each list varied depending on the experimental condition (see Table S1). Thus, the number of relevant words in each list varied between 3 (low memory load) and 5 (high memory load). The number of irrelevant words varied between two (in the low suppression condition) and five (in the high suppression condition). Finally, the number of abstract filler words varied between 2 and 7. Target words (relevant and irrelevant) were familiar concrete nouns referring to body parts, objects, or animals that can be classified by size. Filler words were abstract nouns. Word frequency, concreteness, and familiarity were controlled. The final 24 lists were distributed in 4 experimental conditions of 6 lists. One list of each experimental condition was considered a practice list. The total number of words to be recalled across all condition lists was 80 (practice lists were excluded), 25 in each high memory load condition, and 15 in each low memory load condition. The dependent variable was the proportion of previous-list intrusions. The average task duration was 20 minutes.

**S1 Table. Composition of the lists as a function of the experimental conditions.**

| Low load/low suppression  (5 Lists) | Low load/high suppression  (5 Lists) | High load/low suppression  (5 Lists) | High load/high suppression  (5 Lists) |
| --- | --- | --- | --- |
| 3 Relevant items  2 Irrelevant items  7 Filler items | 3 Relevant items  5 Irrelevant items  4 Filler items | 5 Relevant items  2 Irrelevant items  5 Filler items | 5 Relevant items  5 Irrelevant items  2 Filler items |

**Negative Priming**

The current version of the task is adapted from the previous work of Tipper (1985) and Pritchard & Neumann (2004). These previous studies used the negative priming effect as an index of cognitive inhibition in children (see also Rueda et al., 2005)^[[1]](#footnote-2)^. The stimuli included line drawings from the Snodgrass & Vanderwart (1980)’s corpus (see S6 Fig) representing objects or animals. These displays were organized into pairs consisting of a prime display followed by a probe display. An entirely overlapping red shape (non-target) and green shape (target) in each prime and probe display was revealed at the center of the screen. A black comparison shape appeared randomly, either above or under the overlapping shapes. The area occupied by the overlapping shapes subtended a visual angle of 2.4º x 2.4º, as well as the area occupied by the comparison shape. The distance between the outer edge of the overlapping and comparison shapes was about 0.4º. The red shape was described as a distractor, and participants were advised to ignore it to their best. Participants were then instructed to decide if the green target shape matched the black comparison shape by pressing "Z" and "M" keys with their left and right index fingers for "different" and "same" responses, respectively, as quick as possible. At the bottom of the screen, the words "different" and "same" were displayed as reminders of this key assignment. Half of the experiment trials were control trials (in which the red prime distractor and the black target probe were different items), and half were ignored repetition trials (the red prime distractor and the black target probe were the same items). Each session began with a set of 20 practice trials. Then, information about mean reaction times and hit rate during practice block was displayed on the screen. The experimenter decided if the participant needed to repeat the practice block or was ready to start the experimental phase as a result of his/her performance. The experimental phase consisted of 216 trials, divided into four blocks of 54 each. Each experimental trial consisted of a couplet of prime-probe displays. It began with a 2000 ms presentation of a cross-shape fixation point followed by a blank screen for 500 ms before the prime display. In the prime display, the overlapping red and green shapes and the black comparison shape appeared simultaneously. Participants had to decide which green shape (to be attended) was the same as the black shape pressing the keys "same" or "different." The prime display remained on the screen until response, followed by a 500 ms interval in which a blank screen was displayed before the probe display. The overlapping red and green shapes and the black comparison shape were presented simultaneously and remained until a response in the probe display. In the ignore repetition condition, the stimulus to be ignored in the prime display (red) was the stimulus to be attended in the probe display (green). The stimulus to be ignored in the prime trial (red) did not become the stimulus to be attended (green) in the probe trial in the control condition. Feedback was provided for prime and probe responses only during the practice block. The dependent variable was RT/percentage of hits for the ignore repetition trials in the probe condition. The average task duration was 20 minutes.

**S6 Fig. An example of an ignored repetition prime–probe pair requiring a "same" response both in prime and probe displays.**

| Prime | Probe |
| --- | --- |
|  | 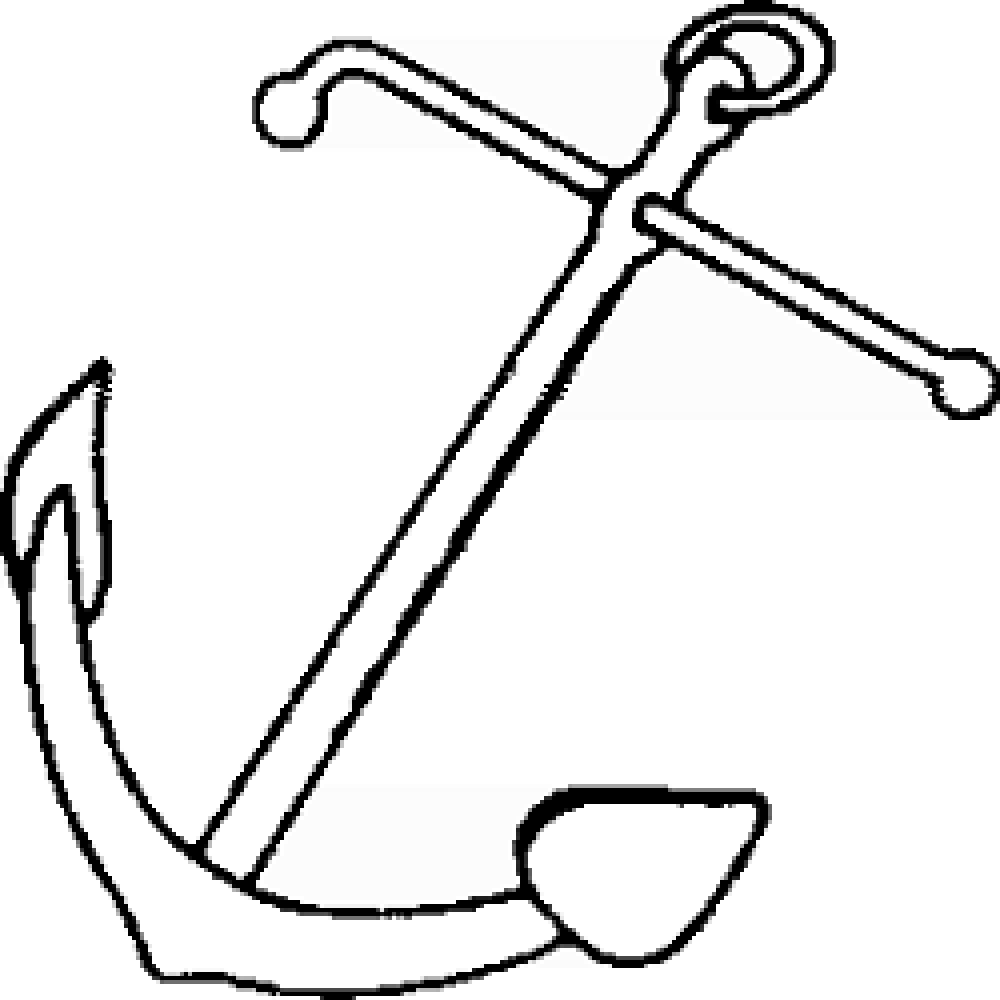 |
| 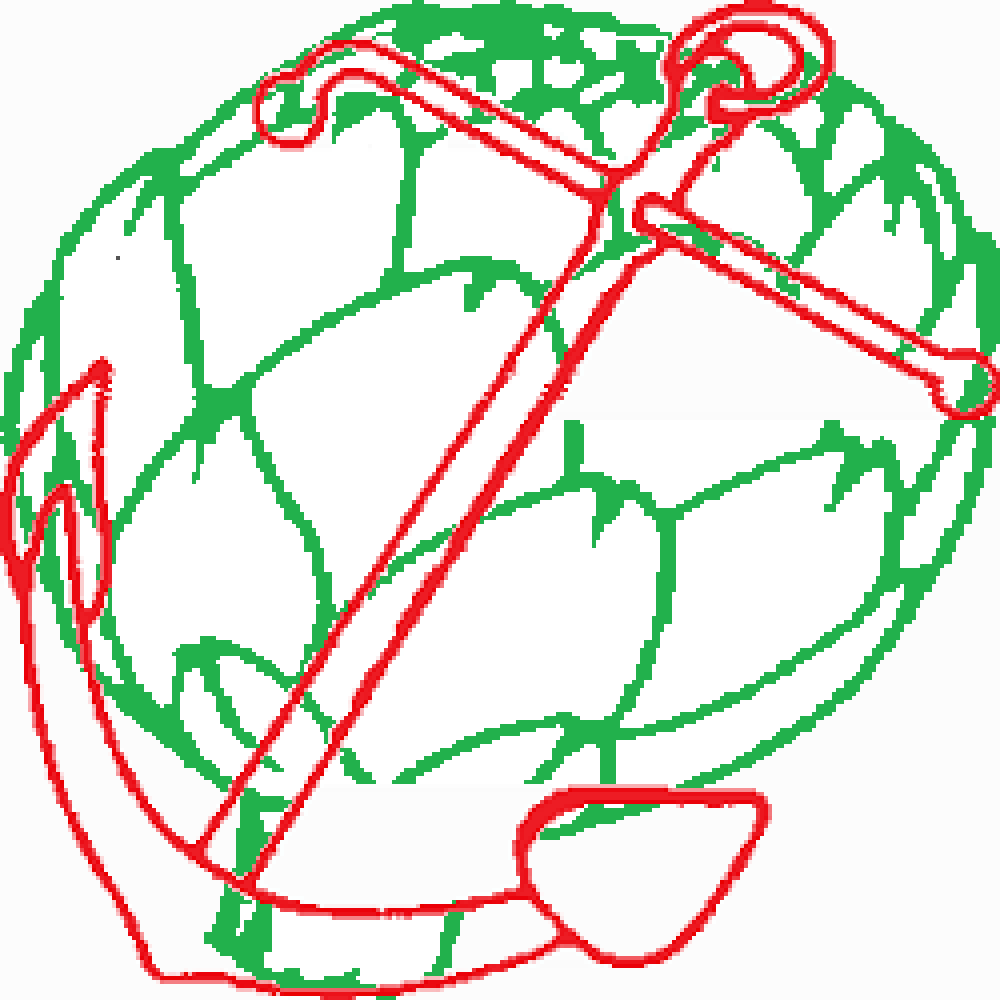 | 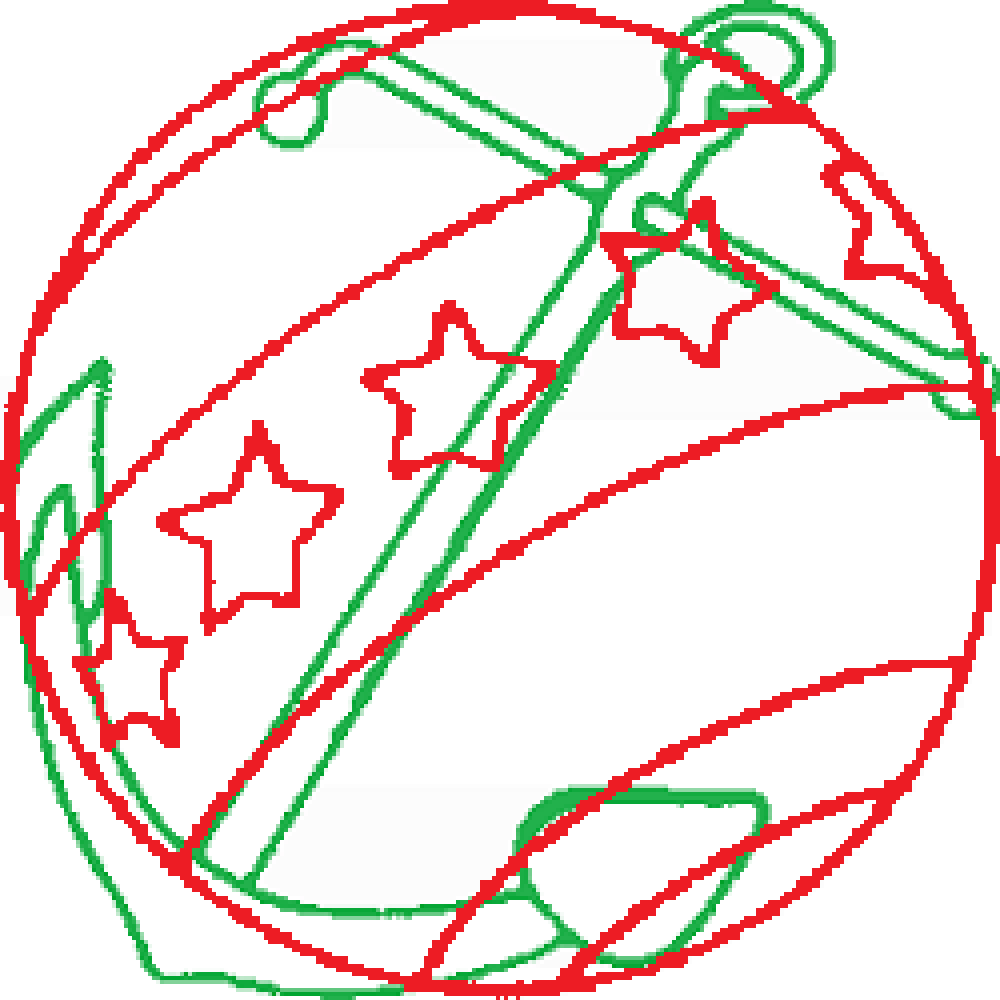 |
| 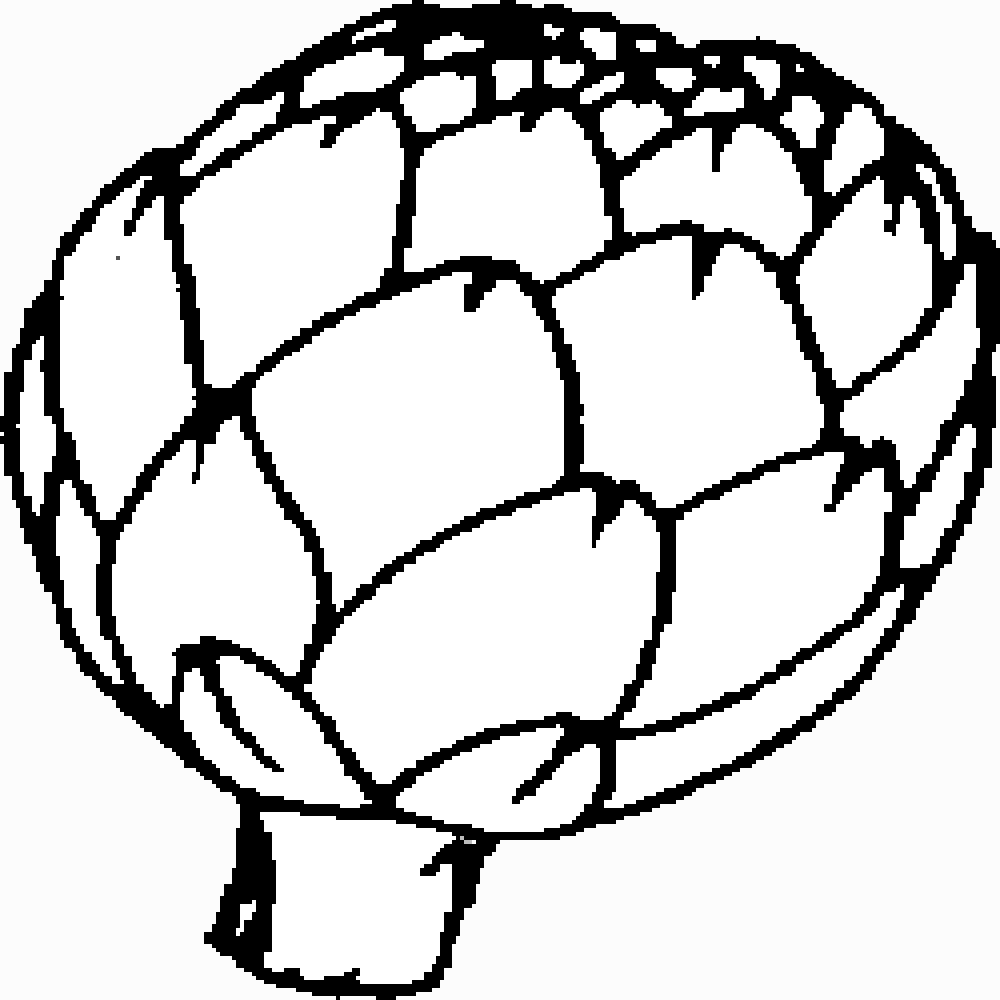 |  |

Note. The comparison shapes on the bottom and the top of each display were black, the target shapes in the center were green, and the distractor shapes in the center were red. Children were asked to respond "same" if the green and black items matched and "different" if they did not match in both displays.

1. **Processing Speed**

We used the RT/percentage of hits in the neutral condition of the Stroop task. This measure has been previously used in a study on the structure and organization of executive functioning across development by (Huizinga et al., 2006).

1. **Working Memory Capacity**

**Reading Span Test for children (PAL-N).**

The current version of the task is an adaptation for children of Daneman & Carpenter's (1980) Reading Span Task (Carriedo & Rucián, 2009). It measures the ability to process and store verbal information simultaneously, with no option to review strategies. Participants had to read aloud each sentence at their own pace and remember the last word of the sentence. The task consisted of 48 phrases (6 training and 42 experimental) grouped into levels of 2, 3, 4, and 5 sentences, with three series of sentences for each level. At the end of each series, participants had to remember the last word of each sentence in the same order as presented. Sentences were presented individually in the center of a computer screen only for the time needed to read the sentence. As soon as the participant finished reading, a new sentence appeared. When the series finished, a question mark appeared on the screen, asking the participant to remember the words. The task ended when the participant was unable to remember any of the series of a given level. The dependent variable was reading span, considered at the level at which the participant had correctly answered at least 2 of the three series. The reliability and validity have been shown through hundreds of studies, ranging from .70–.90 for span scores (see Conway et al., 2005 for a review).  The average task duration was 15 minutes.

**S2 Table. Example of the three series of sentences of level 2. In bold, the word to be remembered in the same order as presented.**

| **Series 1**  Harry Potter is the most famous character in **cinema**  The summer was so cold that many people changed their **plans**  **Series 2**  Yesterday, the whole town listened to the mayor's **speech**  When flying, the pigeon lost a **feather**  **Series 3**  With his beautiful eyes he directed a deep look at his **mother.**  When we saw that he had a fever we went to tell the **doctor** |
| --- |
|  |

**Counting Span Task (CST)**

This task was designed by Case et al., (1982). It measures the ability to process and store nonverbal information simultaneously, with no option to use review strategies. It has the same structure as Daneman and Carpenter's Reading Span Test with the difference that participants had to process visual information (counting and pointing out geometric figures) rather than verbal information. It consists of 48 visual displays (6 training and 42 experimental) grouped into levels. Each level contains 3 series of 2, 3, 4, and 5 displays each. Each display is formed by 18 squares, of which a minimum of 3 and a maximum of 8 are blue, and the rest are red squares to act as distractors (see S7 Fig). Participants had to count the blue squares of each display without a pause. At the end of the series, they had to remember the number of squares counted in the same order in which they were presented. CST, in its original format, was handed out manually. However, we presented the stimuli on a computer screen. Initially, 42 experimental slides and six practice slides were built with 18 red and blue squares on a white background. All squares were randomly placed on each slide regarding location and rotation angle (greater or lesser rotation). The order of presentation of the slides was randomized so that patterns of numbers that facilitated remembrance did not appear in the same series (e.g., 4-5-6; 3-5-7). Each configuration of blue and red squares was presented individually on a computer screen for as long as it took to the participant to count them. When the participant finished counting, a new display appeared on the screen. To prevent repetition strategy, children had to point and count aloud each of the blue squares. A question mark appeared on the screen when the series finished, asking the participant to remember the numbers. The task ended when the participant was unable to remember any of the series of a given level. The reliability and validity have been shown through hundreds of studies, ranging from .70–.90 for span scores (see Conway et al., 2005 for a review). The average task duration was 10 minutes.

**S7 Fig. Example of one series of displays of level 3. Participants had to point out and counting blue squares.**

| 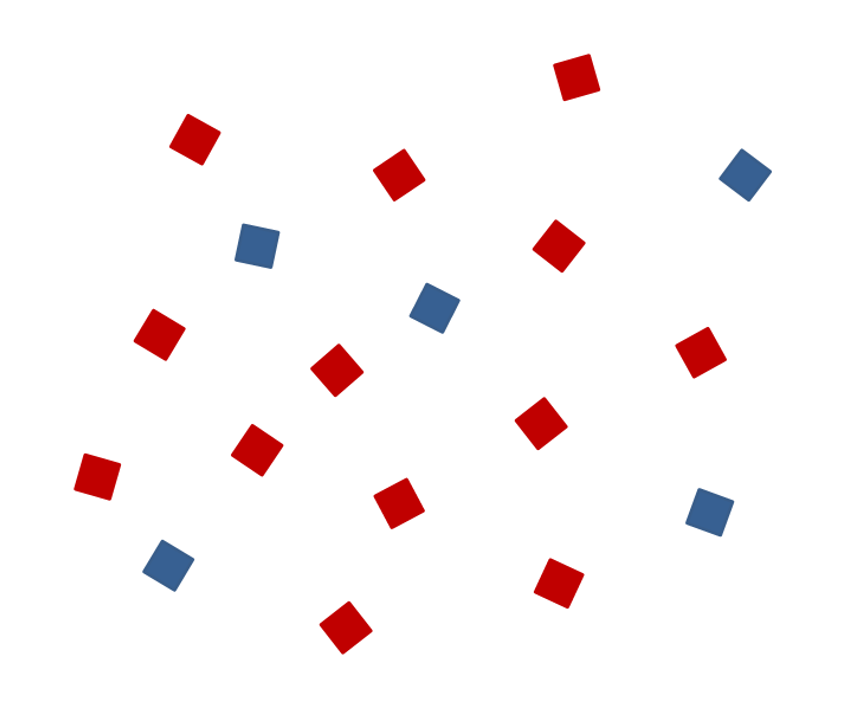 |
| --- |
| 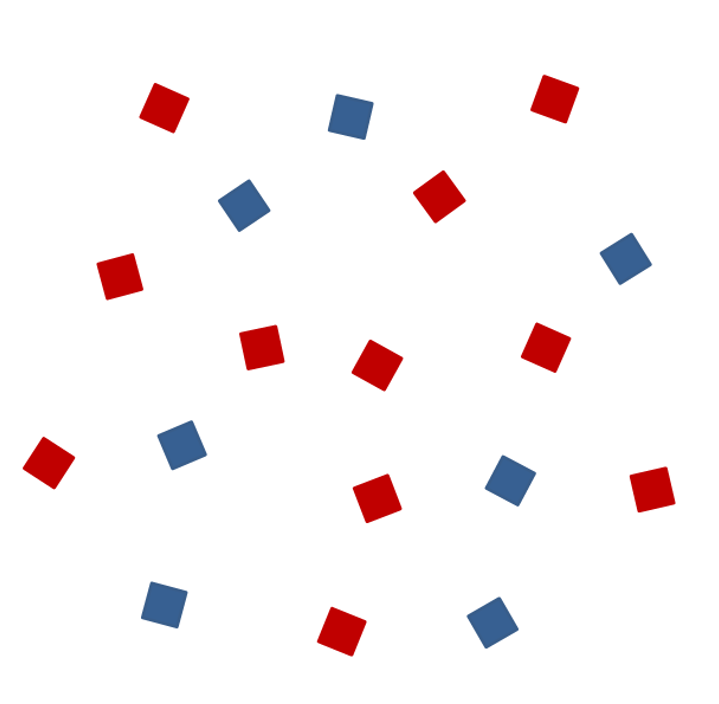 |
| 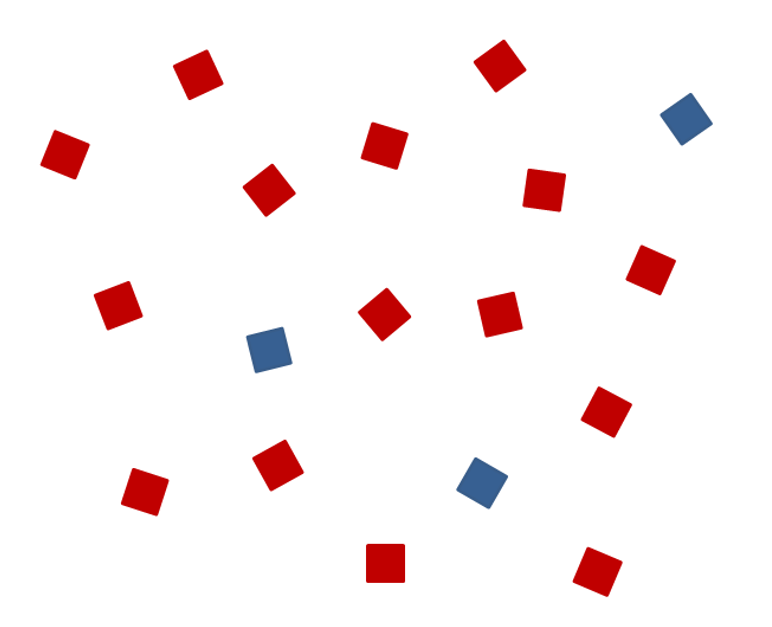 |

**Section B. Preliminary analysis**

**Descriptive Statistics**

Proportions of correct answers and RTs for the inhibition tasks are presented in S3 Table for each age group. The boxplot in S8 Fig provides an overall summary of data distribution for all inhibition measures according to age group. The boxes represent the interquartile range (IQR; 25^th^ to 75^th^ percentile). The horizontal black lines in each box represent the median, the notches show the approximate 95% CI of the median, and the dots inside the boxes denote the location of the mean. The length of whiskers is 1.5*IQR, and data beyond the whiskers' end are plotted individually. All measures were transformed to Z-scores.

**S8 Fig.Box plots for inhibition measures (Z-scores). RT: response time; ACC: accuracy.**

As per S8 Fig, except for the *receptive attention* and the *stop signal tasks*, variability in all inhibition measures decreases as the age of the group increases. The *receptive attention task* and the *stop signal task* showed a similar variation across age groups. Visual inspection of the 95% CIs of the medians suggests that in *flanker, local-global, Stroop*, and *negative priming tasks*, the 7-year-olds showed longer response times than the 11- and 15-year-olds, and the latter group exhibited shorter response times than the former. Concerning the accuracy tasks, the 95% CIs of the medians indicate that in *go-no-go* and intrusions, the performance of the 7-year-olds was not as good as that of the other age groups. In the *receptive attention task*, the 95% CI of the median indicates better performance of the 7-year-olds than the 11-year-olds. However, the performance of the latter group was better than that of the former in the *go-no go* and *updating information in WM (intrusions index) tasks*. The performance on the stop signal task was higher in 7-year-olds group than in the other age groups, although 7- and 11-year-olds showed similar performance.

S9 Fig shows the boxplots for the covariates: *response times* in the neutral condition of the Stroop task (processing speed) and for accuracy in the WM tasks (reading and counting span). In the left panel of the S9 Fig, the neutral condition of the *Stroop task* indicates higher variation and longer response times for age group 7 than for other age groups. Response times in the neutral condition of the *Stroop task* decrease as the age of the group increases. The middle and right panels of S9 Fig indicate that age group 15 exhibited higher variability and better performance than the other age groups in both the WM tasks. Moreover, S9 Fig shows that the WM performance of age group 11 was better than the performance of age group 7.

**S9 Fig. Box plots for speed and WMC measures (Z-scores). RT: response time; ACC: accuracy.**

**S3 Table. Means (and Standard Deviations) for the inhibitory tasks and processing speed (RT/Accuracy) and for Working Memory Tasks.**

|  | **Task*** | **7year-olds** | **11 year-olds** | **15 year olds** | **Anova/Ancova**  **Results** |
| --- | --- | --- | --- | --- | --- |
| **Response-distractor inhibition** | **Flanker** |  |  |  | 7>11>15 |
|  | congruent | 972.59 (242.36) | 624.72(154.33) | 476.38(84.32) |  |
|  | incongruent | 1437.04(435.87) | 774.12(261) | 505.23(112.11) |  |
|  | **Local-Global** |  |  |  | 7>11~15 |
|  | congruent | 983.79(288.44) | 624.21(142.21) | 499.83(82.22) |  |
|  | incongruent | 1143.21(361.19 | 706.47(156) | 558.63(92.56) |  |
|  | **Receptive attention** |  |  |  | 11<15 |
|  | base score | .17(.04) | .16(.05) | .25(.06) |  |
|  | **Go-no go** |  |  |  | 7<11~15 |
|  | correct no go | .79(.11) | .87(.10) | .90(.07) |  |
|  | **Stroop** |  |  |  | 7>11>15 |
|  | congruent | 1235.49(380.41) | 779.03(224.11) | 620.63(158.94) |  |
|  | incongruent | 1571.68(533.18) | 960.48(281.09) | 742.73(225.21) |  |
|  | **Stop signal** |  |  |  | 7>11~15 |
|  | Stop Signal RT | 516(233) | 324 (141) | 332 (158) |  |
| **Cognitive inhibition** | **Updating WM task** |  |  |  | 7>11~15 |
|  | Previous-list intrusions | .24(.15) | .19(.14) | .14(.10) |  |
|  | **Negative Priming** |  |  |  | 7>11>15 |
|  | control | 1485,71(205,01) | 1028,84(223,13) | 790,40(134,26) |  |
|  | ignored | 1403,67(346,21) | 1105,93(240,66) | 859.98(152,70) |  |
| **Processing Speed** | Stroop Neutral | 864 (211) | 610(133) | 517(102) | 7<11<15 |
| **WMC** | RST | 2.57 (.37) | 2.76(.242) | 3.21(.50) | 7<11<15 |
|  | Counting Span | 3.64(.63) | 3.85(.60) | 4.61(.60) | 7<11<15 |
| ***** For interpreting the direction of the differences, it is necessary to take into account that the dependent variable for speeded tasks (flanker, local-global, Stroop, and negative priming) is RT/ACC. Thus, slowest RT’s show lower performance. The dependent variable for updating is errors. The dependent variable for go-no go, RST, and counting span tasks are accuracy | | | | | |

**Correlational analysis**

Correlations and partial correlations among inhibitory tasks and processing speed are shown in S4 - S6 Tables.

**S4 Table. Pearson's r Correlations (bottom half) and partial correlations (top half shadowed) for the seven-year-old group.**

|  | **Flanker** | | **Local-global** | | | **Receptive Attention** | | | **Go-no go** | | | **Stop Signal** | | | **Stroop** | **Intrusions in WM** | | | **Negative Priming** | | | **Counting Span** | | | **RST** | |  |
| --- | --- | --- | --- | --- | --- | --- | --- | --- | --- | --- | --- | --- | --- | --- | --- | --- | --- | --- | --- | --- | --- | --- | --- | --- | --- | --- | --- |
| **Flanker** | — |  | | .10 |  | | .13 |  | | .09 |  | | .15 |  | .16 |  | .18 | * | | .27 | *** | |  |  | |  |  |
| **Local-global** | .19 | * | | — |  | | -.22 | ** | | -.29 | ** | | .00 |  | .12 |  | .07 |  | | .09 |  | |  |  | |  |  |
| **Receptive Attention** | -.13 |  | | -.23 | ** | | — |  | | .25 | ** | | -.20 | * | .07 |  | .25 | ** | | .26 | ** | |  |  | |  |  |
| **Go-no go** | -.13 |  | | -.34 | *** | | .26 | ** | | — |  | | .13 |  | -.05 |  | -.09 |  | | -.20 | * | |  |  | |  |  |
| **Stop Signal** | .10 |  | | -.07 |  | | -.20 | * | | -.01 |  | | — |  | .04 |  | .24 | ** | | .18 | * | |  |  | |  |  |
| **Stroop** | .23 | ** | | .25 | ** | | -.01 |  | | -.12 |  | | .03 |  | — |  | .08 |  | | .11 |  | |  |  | |  |  |
| **Intrusions in WM** | .20 | * | | .10 |  | | -.25 | ** | | -.11 |  | | .22 | * | .11 |  | — |  | | .21 | ** | |  |  | |  |  |
| **Negative Priming** | .32 | *** | | .22 | ** | | -.26 | ** | | -.25 | ** | | .13 |  | .23 | ** | .23 | ** | | — |  | |  |  | |  |  |
| **Counting Span** | -.07 |  | | -.23 | ** | | .12 |  | | .09 |  | | .17 | * | -.00 |  | -.06 |  | | -.06 |  | | — |  | |  |  |
| **RST** | -.13 |  | | -.24 | ** | | -.06 |  | | .09 |  | | .31 | *** | -.06 |  | -.06 |  | | -.13 |  | | .45 | *** | | — |  |
| **Processing Speed** | .22 | ** | | .37 | *** | | -.08 |  | | -.17 | * | | -.02 |  | .42 | *** | .08 |  | | .34 | *** | | -.19 |  | | -.13 |  |

Note. * p < .05, ** p < .01, *** p < .001

**S5 Table. Pearson's r Correlations (bottom half) and partial correlations (top half shadowed) for the 11-year-old group.**

|  | **Flanker** | | **Local-global** | | **Receptive Attention** | | **Go-no go** | | **Stop Signal** | | **Stroop** | | **Intrusions in WM** | | **Negative Priming** | | **Counting Span** | | **RST** | |
| --- | --- | --- | --- | --- | --- | --- | --- | --- | --- | --- | --- | --- | --- | --- | --- | --- | --- | --- | --- | --- |
| **Flanker** | — |  | .37 | *** | -.31 | *** | -.23 | ** | .24 | ** | .33 | *** | .06 |  | .17 | * |  |  |  |  |
| **Local-global** | .51 | *** | — |  | -.26 | ** | -.25 | ** | .15 |  | .44 | *** | .08 |  | .11 |  |  |  |  |  |
| **Receptive Attention** | -.41 | *** | -.36 | *** | — |  | .07 |  | -.26 | ** | -.19 | * | .05 |  | .20 | * |  |  |  |  |
| **Go-no go** | -.28 | *** | -.30 | *** | .11 |  | — |  | -.30 | *** | -.09 |  | -.04 |  | -.06 |  |  |  |  |  |
| **Stop Signal** | .28 | *** | .20 | * | -.29 | *** | -.31 | *** | — |  | .24 | ** | .06 |  | .27 | *** |  |  |  |  |
| **Stroop** | .46 | *** | .57 | *** | -.27 | *** | -.16 |  | .27 | *** | — |  | .11 |  | .20 | * |  |  |  |  |
| **Intrusions in WM** | .18 | * | .19 | * | -.04 |  | -.05 |  | .10 |  | .20 | * | — |  | .27 | *** |  |  |  |  |
| **Negative Priming** | .28 | *** | .23 | ** | -.26 | ** | -.11 |  | .29 | *** | .27 | *** | .32 | *** | — |  |  |  |  |  |
| **Counting Span** | -.38 | *** | -.32 | *** | .24 | ** | .18 | * | -.08 |  | -.21 | ** | -.20 | * | -.29 | *** | — |  |  |  |
| **RST** | -.36 | *** | -.29 | *** | .30 | *** | .05 |  | -.13 |  | -.18 | * | -.20 | * | -.16 |  | .48 | *** | — |  |
| **Processing Speed** | .37 | *** | .44 | *** | -.21 | * | -.13 |  | .13 |  | .55 | *** | .18 | * | .18 | * | -.26 | ** | -.33 | *** |

Note. * p < .05, ** p < .01, *** p < .001

**S6 Table. Pearson's r Correlations (bottom half) and partial correlations (top half shadowed) for the 15-year-old group.**

|  | **Flanker** | | **Local-global** | | **Receptive Attention** | | **Go-no go** | | **Stop Signal** | | **Stroop** | | **Intrusions in WM** | | **Negative Priming** | | **Counting Span** | | **RST** |
| --- | --- | --- | --- | --- | --- | --- | --- | --- | --- | --- | --- | --- | --- | --- | --- | --- | --- | --- | --- |
| **Flanker** | — |  | .47 | *** | -.24 | ** | -.25 | ** | .16 |  | .14 |  | .23 | ** | -.02 |  |  |  |  |
| **Local-global** | .51 | *** | — |  | -.21 | * | -.29 | *** | .35 | *** | .25 | ** | .31 | *** | .10 |  |  |  |  |
| **Receptive Attention** | -.32 | *** | -.28 | *** | — |  | .07 |  | -.19 | * | -.06 |  | -.13 |  | -.15 |  |  |  |  |
| **Go-no go** | -.15 |  | -.20 | * | .02 |  | — |  | -12 |  | -.05 |  | -.07 |  | -.02 |  |  |  |  |
| **Stop Signal** | .21 | * | .38 | *** | -.22 | ** | -.10 |  | — |  | .19 | ** | .36 | *** | .04 |  |  |  |  |
| **Stroop** | .35 | *** | .41 | *** | -.20 | * | .07 |  | .24 | ** | — |  | .14 |  | .04 |  |  |  |  |
| **Intrusions in WM** | .31 | *** | .38 | *** | -.19 | * | -.04 |  | .40 | *** | .27 | *** | — |  | .07 |  |  |  |  |
| **Negative Priming** | .02 |  | .14 |  | -.17 | * | -.01 |  | .07 |  | .10 |  | .11 |  | — |  |  |  |  |
| **Counting Span** | -.02 |  | -.08 |  | .04 |  | .09 |  | -.10 |  | -.08 |  | -.16 |  | -.09 |  | — |  |  |
| **RST** | -.16 |  | -.10 |  | .09 |  | .08 |  | -.13 |  | -.19 | * | -.20 | * | -.08 |  | .57 | *** | — |
| **Processing Speed** | .41 | *** | .35 | *** | -.25 | ** | .20 | * | .13 |  | .59 | *** | .22 | ** | .09 |  | .09 |  | -.07 |

Note. * p < .05, ** p < .01, *** p < .001

**ANCOVA Analysis**

ANOVAs were computed to test age-related changes in covariates, with the age group as a between-group variable. Moreover, several ANCOVAS were carried out on each inhibitory task, with age group as a between-group variable, condition as a within-subjects variable (only for timing tasks in which congruent and incongruent conditions are presented), and processing speed and/or WMC as covariates.

#### *Processing Speed*

The ANOVA showed a significant effect of age, *F*(2, 447) = 200, *p* < .001, *η^2^* = .47. Bonferroni comparisons showed a significant decrement of processing speed from 7-year-olds to 15-year-olds (all *p*s <.001).

#### *Working Memory Capacity*

The ANOVA showed a significant effect of age both for *RST*, *F*(2, 447) = 83.8, *p* < .001, *η*^2^ = .27, and *counting span*, *F*(2, 447) = 105, *p* < .001, *η*^2^ = .32. Bonferroni comparisons showed that WMC significatively increased from 7-year-olds to 15-year-olds (all *p*s <.01) for both WMC tasks.

#### *Distractor Inhibition*

*Flanker Task.* The ANCOVA showed that only the covariate processing speed, *F*(1, 444) = 46.56, *p* < .001, η^2^ = .010 was significantly related to the participant performance. Reading Span Test was also significant, *F*(1, 444) = 3.77, *p* < .05, η^2^ = .008. There was also a significant effect of age, *F(*2, 444) = 11.59, *p* < .001, η^2^ = .02, condition (congruent vs. incongruent) *F(*1, 444) = 1.62, *p* < .001, η^2^ = .02, and the interaction age x condition *F(*2, 444) = 49.65, *p* < .001 η^2^ = .18, after controlling for the effect of processing speed and WMC. Post hoc Bonferroni comparisons showed significant differences among 7-year-olds and 11 and 15-year-olds (all *p’s* < .001), Also, the difference between congruent and incongruent trials decreased significatively with age in the expected direction (all *p*s < .05, one-tailed).

*Local-global Task.* The ANCOVA showed that both covariates processing speed and WMC (counting span) were significantly related to the participant’s performance [processing speed, *F*(1, 444) = 103.30, *p* < .001, η^2^ = .19, and counting span, *F*(1, 444) = 1.59, *p* < .001, η^2^ = .02] . There was also a significant effect of age, *F(*2, 444) = 53.53, *p* < .001, η^2^ = .19, condition (compatible vs. incongruent), *F(*1, 444) = 1.01, *p* < .001, η^2^ = .02, and the interaction age x condition, *F(*2, 444) = 7.36, *p* < .001, η^2^ = .03, after controlling for the effect of processing speed and WMC. Bonferroni comparisons showed significant differences among 7-year-olds and 11 and 15-year-olds (all *p’s* < .001), but not between 11 and 15-year-olds. Also, differences between compatible and incongruent conditions were significant in all age groups (all *p’s* <.001).

*Receptive Attention Task.* This task has subtle differences for 7-year-olds and 11 and 15-year-olds. Thus, only the performance of 11 and 15-year-olds was compared. Moreover, as the *base score* used as a dependent variable is already corrected for speed (see Naglieri & Das, 1997), we only corrected WMC. We found a significant effect of WMC (RST) covariate, *F(*2, 296) = 7.36, *p* < .001, η^2^ = .03. We also found a significant effect of age after controlling for WMC, *F(*1, 296) = 113.11, *p* < .001, η^2^ = .28. The performance of 11-year-old children was worse than those of 15-year-olds.

#### *Response inhibition*

*Go-no Go Task.* The ANCOVA showed a significant effect of the covariate WMC (Counting Span), *F*(1, 445) = 4.41, *p* < .05, *η*2 = .01. Moreover, age had a significant effect, *F*(2, 445) = 3.37, *p* < .001, *η*2 = .12. Bonferroni comparisons showed significant differences among seven-year-olds and the rest of the groups, but not between 11 and 15-year-olds after controlling for WMC (all ps <.001).

*Stop-signal Task. I*n this case, we used only WMC as a covariate because the stop signal delay is adjusted continuously with the staircase tracking procedure, which is timing dependent. The ANCOVA did not show a significant effect of WMC, but it did show a significant effect of age, *F*(2, 445) = 47.03, *p* < .001, *η*2 = .2517. Bonferroni comparisons showed that the time for stopping response was larger for 7-year-olds than for 11 and 15-year-olds (all *p*s < .001), but it was mostly the same for 11-year-olds and 15-year-olds.

*Stroop task.* The ANCOVA showed that the covariate processing speed was significantly related to the participant’s performance, *F*(1, 444) = 158.77, *p* < .001, *η*2 = .26. There was also a significant effect of age, *F*(2, 444) = 32.21, *p* < .001, *η*2 = .13, condition (compatible vs. incongruent), *F*(1, 445) = 3.89, *p* < .05, *η*2 = .009, and the interaction age x condition, *F*(2, 446) = 9.32, *p* < .001, *η*2 = .04. Post hoc Bonferroni comparisons showed significant differences among all ages and that the difference between compatible and incongruent trials decreased with age in the expected direction (all *p*s < .001) after controlling for the effect of processing speed.

#### *Cognitive inhibition*

*Intrusions in WM.* The ANCOVA showed a significant effect of WMC (RST), *F(*1, 445) = 4.10, *p* < .05, η^2^ = .0093. Moreover, we found a significant effect of age, *F(*2, 445) = 7.04, *p* < .001, η^2^ = .03. Bonferroni comparisons showed that the proportion of intrusions was larger for 7-year-olds than for 11-year-olds and 15-year-olds (all *p*s <.01), but differences between 11 and 15-year-olds were not significant after controlling WMC.

*Negative Priming.* The ANOVA showed a significant effect of both covariates: processing speed, *F(*1, 444) = 37.79, *p* < .001, η^2^ = .07, and WMC (Counting Span), *F(*1, 444) = 5.97, *p* < .05, η^2^ = .01. Moreover, the effect of age, *F(*2, 444) = 92.09, *p* < .001, η^2^ = .29, and condition (control vs ignored), *F(*1, 444) = 6.70, *p* < .01, η^2^ = .02, was also significant. Bonferroni comparisons showed significant differences among all age groups and between ignored and control conditions after controlling for the effect of processing speed and Counting Span (all *p’s* < .001).

ANCOVA results showed that, within each age group, all differences between control and interference conditions for flanker, local-global, Stroop, and negative priming were significant (all *p's* <.001), which means that the tasks were sensitive to discriminate between experimental conditions across ages.

**Section C. Multi-Group Confirmatory Factor Analysis (main analysis)**

**S10 Fig. Two-factor model.**

Response distractor: response distractor factor; Cognitive inhibition: cognitive inhibition factor; Stroop neutral: response time for neutral condition of Stroop task; flanker: response time/accuracy for incongruent condition of flanker task; local-global: response time/accuracy for incongruent condition of local-global task; DN (receptive attention): accuracy; go no-go: accuracy no go trials; Stroop: response time/accuracy for incongruent condition of the Stroop task; stop-signal: SSRT stop signal reaction time; intrus.: number of words incorrectly recalled in updating WM task; negative priming: response time/accuracy for the ignored condition of the negative priming task.

**S11 Fig. One-factor model.**


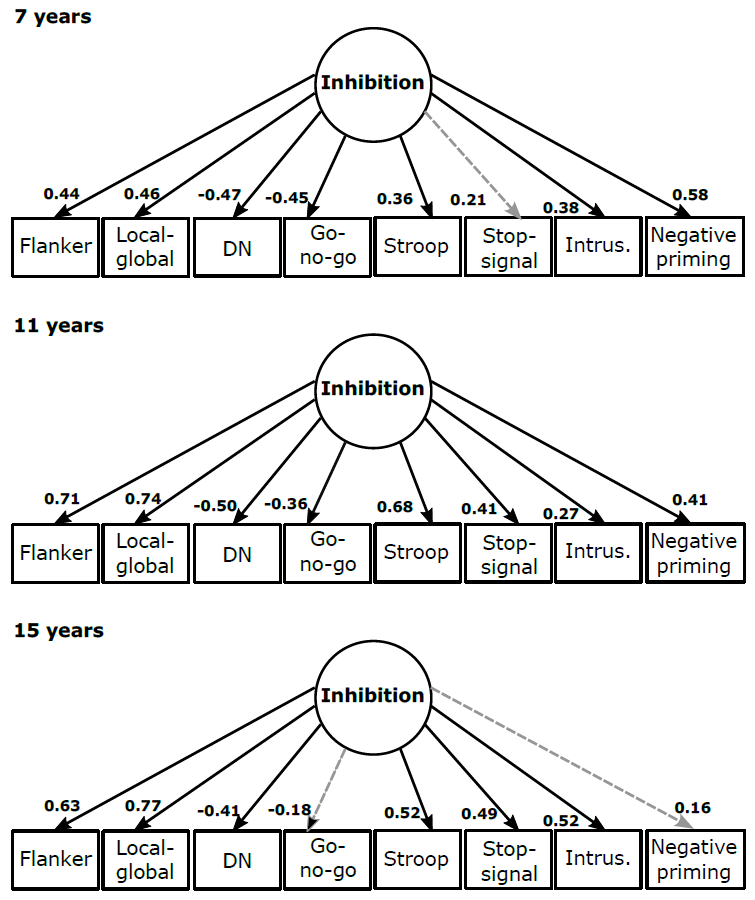


Dashed-gray line indicates non-significant factor loadings (i.e., p > 0.05). Inhibition: inhibition factor; Stroop neutral: response time for neutral condition of Stroop task; flanker: response time/accuracy for incongruent condition of flanker task; local-global: response time/accuracy for incongruent condition of local-global task; DN (receptive attention): accuracy; go-no-go: accuracy no go trials; Stroop: response time/accuracy for incongruent condition of the Stroop task; stop-signal: SSRT stop signal reaction time; intrus.: number of words incorrectly recalled in the updating WM task; negative priming: response time/accuracy for the ignored condition of the negative priming task; Reading span: reading span for Reading span task; counting span: counting span for counting span task.

**S12. Fig. Schematic representation restrictions in the speed factor across age groups.**

M1: model in which factor loadings of inhibition tasks on processing speed were restricted to zero in 7-years group; M2: the same as M1 for the 11-years group; M3: the same as M1 for the 15-years group. Gray lines represent parameters constrained to zero. Speed: processing speed factor; inhibition: inhibition factor; Working memory: WMC factor: reading span task and counting span tasks.; speed factor: Stroop neutral: response time for neutral condition of Stroop task; inhibition factor: flanker: response time/accuracy for incongruent condition of flanker task; local-global: response time/accuracy for incongruent condition of local-global task; DN (receptive attention): accuracy; go no-go: accuracy no go trials; Stroop: response time/accuracy for incongruent condition of the Stroop task; stop-signal: SSRT stop signal reaction time; intrus.: number of words incorrectly recalled in the updating WM task; negative priming: response time/accuracy for the ignored condition of the negative priming task.

**S13 Fig. WM-Speed-Inhibition restricted model.**

*
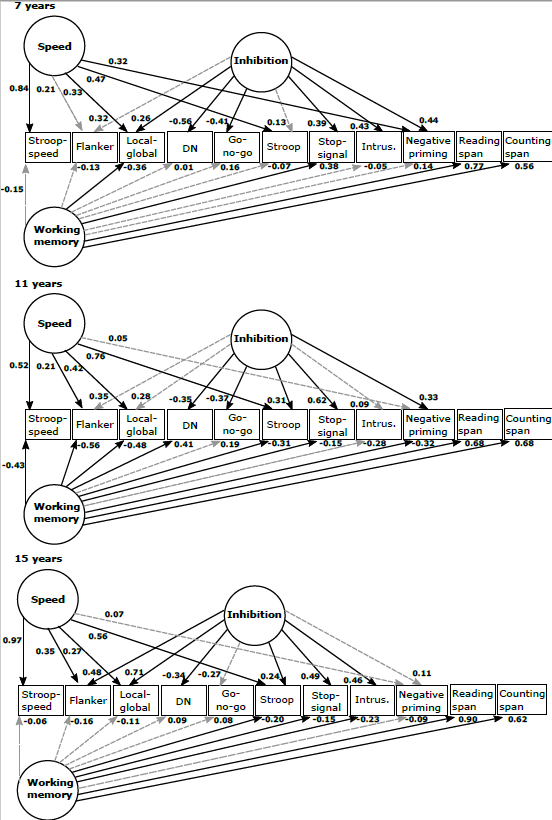
*

Gray lines represent parameters constrained to zero. Speed: processing speed factor; inhibition: inhibition factor; Working memory: WMC factor: reading span task and counting span tasks.; speed factor: Stroop neutral: response time for neutral condition of Stroop task; inhibition factor: flanker: response time/accuracy for incongruent condition of flanker task; local-global: response time/accuracy for incongruent condition of local-global task; DN (receptive attention): accuracy; go no-go: accuracy no go trials; Stroop: response time/accuracy for incongruent condition of the Stroop task; stop-signal: SSRT stop signal reaction time; intrus.: number of words incorrectly recalled in the updating WM task; negative priming: response time/accuracy for the ignored condition of the negative priming task.

**Likelihood Ratio Test for Exploring the Influence of the WMC and Processing Speed for Solving Inhibition Tasks.**

The LRT was employed for comparing the relative fit of nested models. In our case, all restricted models (i.e., M1, M2, ...M6) are nested in the WMC-speed-inhibition model. LRT allows us to examine whether the imposition of restrictions (e.g., speed) in one specific age group (e.g., 7-year-old group) significatively decreases the model fit (e.g., M1) with respect to the WMC-speed-inhibition model. The comparison in fit between one restricted model (e.g., M1) against the WMC-speed-inhibition model helps us to understand, uniquely, the relative importance of speed in one specific age at time (e.g., 7-year-olds group) but it does not allow to compare the relevance of speed or WMC *between* age groups. Thus, for examining the role of processing speed and WMC between age groups, we used the AIC as model selection tool. Specifically, we employed the ΔAIC between the model with the smallest value and other candidate models in the set to discriminate between models. ΔAIC between 0 and 2 suggests limited support to distinguish between models; from 4 to 7 indicates less support for the model with the higher value. A difference > 10 indicates no support for the model with the higher value (Burnham & Anderson, 2002).

S7 Table displays the χ^2^ statistic, the degree of freedom, the χ^2^ statistic differences, the degree of freedom differences, and the p-value for each comparison. In the table, the WMC-speed-inhibition model was compared against each restricted model (i.e., M1, M2, M3, M4, M5, M6). The LRT indicates a significant decrease in model fit due to restrictions on speed for the three age groups (see the p-values for models M1, M2, and M3). Regarding the effect of the constraints of inhibition tasks on WMC, the LR test suggests a statistically significant decrease in model fit for the 7- and 11-year-old groups (see the *p*-values for models M4 and M5). The decrease in model fit for the 15-year-olds group was not statistically significant (see the *p*-values for model M6). This suggests that the role of WMC on inhibition tasks was not relevant to the latter age group.

**S7 Table. Likelihood ratio test between the WMC-speed-inhibition model and each restricted model.**

| Model name | χ^2^ | | *df* | | χ^2^ *diff* | | *df-diff* | | *p* | |
| --- | --- | --- | --- | --- | --- | --- | --- | --- | --- | --- |
| WMC-speed-inhibition model vs. M1, M2, M3, M4, M5, and M6 | | | | | | | | | | |
| WMC-speed-inhibition model | | 113.44 | | 90 | | - | |  | | - |
| M1 (7-years, Speed constrained to 0) | | 139.76 | | 94 | | 18.797 | | 4 | | 0.000 |
| M2 (11-years, Speed constrained to 0) | | 135.84 | | 94 | | 34.861 | | 4 | | 0.000 |
| M3 (15-years, Speed constrained to 0) | | 146.37 | | 94 | | 16.457 | | 4 | | 0.002 |
| M4 (7-years, WMC constrained to 0) | | 148.38 | | 98 | | 30.291 | | 8 | | 0.000 |
| M5 (11-years, WMC constrained to 0) | | 160.05 | | 98 | | 33.85 | | 8 | | 0.000 |
| M6 (15-years, WMC constrained to 0) | | 122.25 | | 95 | | 3.669 | | 5 | | 0.597 |

*Note*. χ^2^: the χ^2^ statistic*; df*: degree of freedom; χ^2^ diff: the χ^2^ statistic differences; *df-diff*: degree of freedom differences; *p*: the p-value.

**Section D****. Multi-Group Confirmatory Factor Analysis with neutral conditions as measure of processing speed**

We tested three different models that used the neutral or control conditions as measured variables for the Speed factor. These models aimed to separate inhibition from task-specific variance, processing speed, and working memory. Consequently, subsequent models allowed correlations between the incompatible/incongruent conditions and the neutral/control conditions from the Flanker, Local-Global, Stroop, and Negative Priming tasks.

S14 Fig depicts the Inhibition and Speed model (N). In this model, correlations between the factors were moderate for the 7-year group and strong for the 11-15-year groups. For the 7-year group, all factor loadings for the Speed factor were moderate to strong and were reliable (p < 0.05). The Inhibition factor exhibited unreliable factor loadings for the Flanker, Stroop, and Stop-signal measures. The remaining measures demonstrated low or moderate factor loadings.

In the 11-year group, the Speed factor did not significantly predict the incompatible/incongruent conditions for the Flanker, Local-Global, and Stroop measures. Likewise, the Inhibition factor displayed unreliable loadings for incompatible/incongruent conditions of latter tasks, as well as for Negative priming.

For the 15-year group, two of eight indicators (Local-Global and Negative Priming) did not load onto the Speed factor, while only three of eight indicators (DN, Stop-signal, and Intrusions) did load on the Inhibition factor. Despite the model's good fit to the data, the strong correlations between Speed and Inhibition factors in the 11-15-year groups suggest a lack of clear differentiation between inhibition and general processing speed.

**S14 Fig. Inhibition and Speed Model (N).**


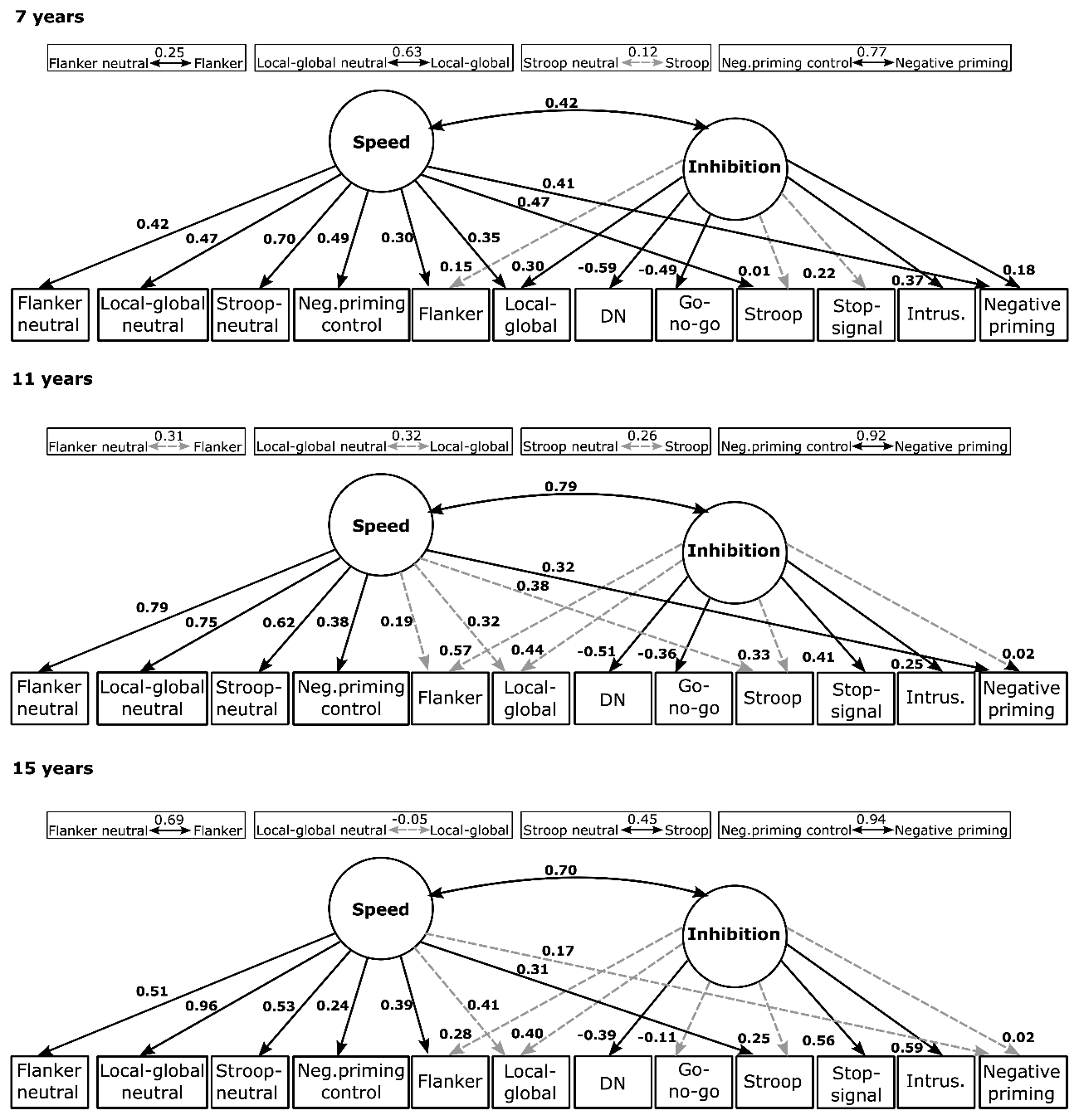


Speed: processing speed factor; Inhibition: inhibition factor; Flanker neutral: response time/accuracy for neutral condition of flanker task; local-global neutral: response time/accuracy for neutral condition of the local-global task; Stroop-neutral: response time for neutral condition of Stroop task; negative priming control: response time/accuracy for control condition of the negative priming task; flanker: response time/accuracy for incongruent condition of flanker task; local-global: response time/accuracy for incongruent condition of Local-global task; DN: accuracy; go-no-go: accuracy no go trials; Stroop: response time/accuracy for incongruent condition of the Stroop task; stop-signal: reaction time to stop signal (RTSS); intrus.: number of errors; negative priming: response time/accuracy for ignored condition of the negative priming task.

S15 Fig presents a model that attempts to disentangle the strong relationships between Speed, Inhibition, and Working Memory (WM) by controlling for individual differences in WM capacity. While the model exhibited an excellent fit (S8 Table), the only significant reduction in correlation was observed in the 11-year group (from .79 to .57).

For the 7-year group, all measures loaded on the Speed factor, while six of eight measures loaded onto the Inhibition factor. For the 11-year group, only three of eight measures (neutral conditions for Flanker, Local-Global, and Stroop tasks) significatively load on the Speed factor. Additionally, only two of eight factor loadings were reliable in the Inhibition factor. In this group, most indicators significantly loaded onto the WM factor. For the 15-years group, three measures (incongruent conditions for Local-Global and Stroop, and Negative priming) did not load on the Speed factor, and only three of eight measures loaded onto the Inhibition factor. Beyond the Span tasks, only the Intrusions measure significantly loaded onto the WM factor for this age group.

Thus, data analysis for the latter model reveals that, after controlling for individual differences in WM, we were unable to separate task-specific and speed-of-processing variance from inhibition. First, the high correlations between neutral/control and incongruent/ignored conditions for Flanker (15 years), Local-Global (7 years) and Negative Priming (all groups) tasks suggest that the latent variables failed to capture this variance. Second, for the 11-15-year groups, the pattern of factor loadings, where most inhibition measures did not load onto the Speed and/or Inhibition factors, hindered our ability to discern the underlying processes of these latent variables. Taken together, we conclude that the introduction of the neutral/control conditions as measures for processing speed did not adequately separate the different sources of variance.

**S15 Fig. Inhibition, Speed, and WM model (N).**


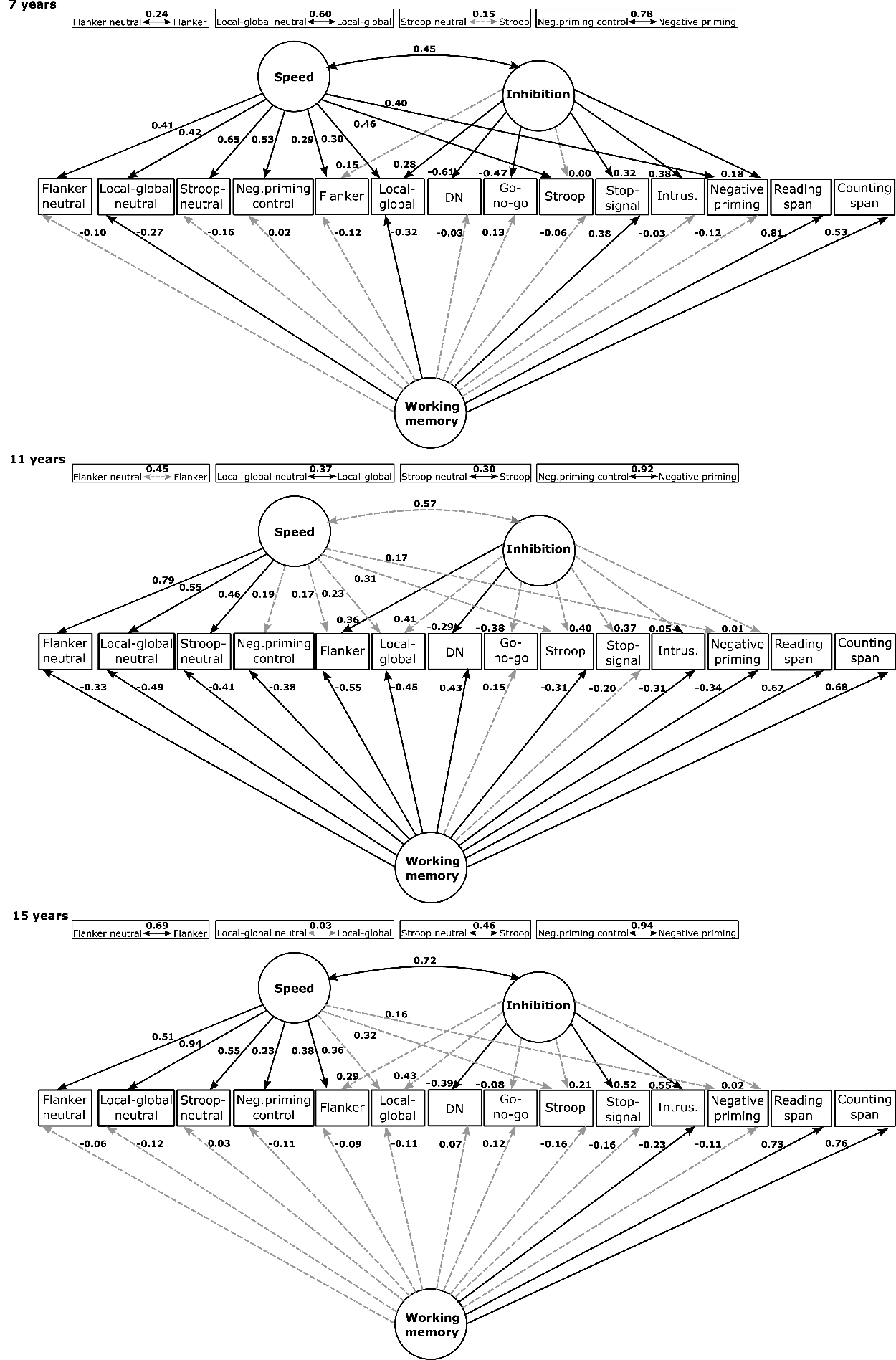


Speed: processing speed factor; Inhibition: inhibition factor; Flanker neutral: response time/accuracy for neutral condition of flanker task; local-global neutral: response time/accuracy for neutral condition of the local-global task; Stroop-neutral: response time for neutral condition of Stroop task; negative priming control: response time/accuracy for control condition of the negative priming task; flanker: response time/accuracy for incongruent condition of flanker task; local-global: response time/accuracy for incongruent condition of Local-global task; DN: accuracy; go-no-go: accuracy no go trials; Stroop: response time/accuracy for incongruent condition of the Stroop task; stop-signal: reaction time to stop signal (RTSS); intrus.: number of errors; negative priming: response time/accuracy for ignored condition of the negative priming task.

S8 Table presents the Goodness-of-Fit indices for the three models that used the neutral or control conditions as measured variables for the Speed factor. All three models demonstrated excellent fit. These models aimed to separate inhibition from task-specific variance, processing speed, and working memory. Consequently, subsequent models allowed correlations between the incompatible/incongruent conditions and the neutral/control conditions from the Flanker, Local-Global, Stroop, and Negative Priming tasks.

**S8 Table. Goodness-of-fit of the neutral condition’s models.**

| Model | YB χ2 | *df* | *p* | Robust CFI | Robust RMSEA |
| --- | --- | --- | --- | --- | --- |
| Inhibition and Speed model (N) | 218.39 | 135 | < .001*** | 0.97 | 0.057[0.037 - 0.074] |
| Inhibition, Speed, and WM model (N) | 251.63 | 168 | < .001*** | 0.98 | 0.047[0.026 - 0.063] |
| Note. Robust CFI: the robust comparative fit index; df: degree of freedom; the robust RMSEA: the robust root means square error approximation; YB χ2: Yuan-Bentler correction factor for the χ2. The 90% confidence intervals of the robust RMSEA are given in brackets. | | | | | |

**Section E****. Multi-Group Confirmatory Factor analysis with difference scores**

S9 Table presents the goodness-of-fit indices for the three models with difference scores. Unfortunately, all three models exhibited significant issues. The Three-factor model (D), the Two-factor model (D), and the One-factor model (D) demonstrated poor fit to the data, while the Three-factor model (D) and WM-Speed-Inhibition model (D) encountered estimation problems, including a non-positive definite covariance matrix for the latent variables and negative variances. Attempts to address these issues through variance restrictions were unsuccessful. Consequently, none of these models are suitable for reliable interpretation. S9 Table shows fit indices for these models. For interested readers R scripts for these models are available at:

<https://osf.io/7f25r/?view_only=ab4999740e364c67a7f7494968753141>

**S9 Table. Goodness-of-fit of the models with difference scores.**

| Model | YB χ2 | *df* | *p* | Robust CFI | Robust RMSEA |
| --- | --- | --- | --- | --- | --- |
| Three-factor model (D) | 73.06 | 51 | .023* | 0.84 | 0.052[0.02 - 0.077] |
| Two-factor model (D) | 74.75 | 57 | .057 | 0.87 | 0.046[0 - 0.072] |
| One-factor model (D) | 75.06 | 60 | .091 | 0.89 | 0.041[0 - 0.068] |
| WM-Speed-Inhibition model (D) | 113.58 | 90 | .048* | 0.94 | 0.039[0.005 - 0.059] |
| Note. Robust CFI: the robust comparative fit index; df: degree of freedom; the robust RMSEA: the robust root means square error approximation; YB χ2: Yuan-Bentler correction factor for the χ2. The 90% confidence intervals of the robust RMSEA are given in brackets | | | | | |

**References**

Burnham, K. P., & Anderson, D. R. (2002). *Model Selection and Multimodel Inference: A Practical Information-Theoretic Approach*. Springer-Verlag. http://sutlib2.sut.ac.th/sut_contents/H79182.pdf

Carriedo, N., Corral, A., Montoro, P. R. P. R., Herrero, L., Ballestrino, P., & Sebastián, I. (2016). The development of metaphor comprehension and its relationship with relational verbal reasoning and executive function. *PLoS ONE*, *11*(3). https://doi.org/10.1371/journal.pone.0150289

Carriedo, N., Corral, A., Montoro, P. R. P. R., Herrero, L., & Rucián, M. (2016). Development of the updating executive function: From 7-year-olds to young adults. *Developmental Psychology*, *52*(4), 666–678. https://doi.org/10.1037/dev0000091

Carriedo, N., Rodríguez-Villagra, O. A., Pérez, L., & Iglesias-Sarmiento, V. (2024). Executive functioning profiles and mathematical and reading achievement in Grades 2, 6, and 10. *Journal of School Psychology*, *106*(May 2023). https://doi.org/10.1016/j.jsp.2024.101353

Carriedo, N., & Rucián, M. (2009). Adaptación para niños de la prueba de amplitud lectora de Daneman y Carpenter (PAL-N). Adaptation of Daneman and Carpenter’s Reading Span Test (PAL-N) for children. *Infancia y Aprendizaje / Journal for the Study of Education and Development*, *32*(3), 449–465. https://doi.org/10.1174/021037009788964079

Case, R., Kurland, D. M., & Goldberg, J. (1982). Operational efficiency and the growth of short-term memory span. *Journal of Experimental Child Psychology*, *33*(3), 386–404. https://doi.org/10.1016/0022-0965(82)90054-6

Christ, S. E., Steiner, R. D., Grange, D. K., Abrams, R. A., & White, D. A. (2006). Inhibitory control in children with phenylketonuria. *Developmental Neuropsychology*, *30*(3), 845–864. https://doi.org/10.1207/s15326942dn3003_5

Conway, A. R. A., Kane, M. J., Bunting, M. F., Hambrick, D. Z., Wilhelm, O., & Engle, R. W. (2005). Working memory span tasks: A methodological review and user’s guide. *Psychonomic Bulletin and Review*, *12*(5), 769–786. https://doi.org/10.3758/BF03196772

Daneman, M., & Carpenter, P. (1980). Individual Differences in Working Memory and Reading. *Journal of Verbal Learning and Verbal Behavior, 1*, *19*, 450–466. https://doi.org/doi:10.1016/S0022-5371(80)90312-6

De Beni, R., & Palladino, P. (2004). Decline in working memory updating through ageing: Intrusion error analyses. *Memory*, *12*(1), 75–89. https://doi.org/10.1080/09658210244000568

Durston, S., Thomas, K. M., Yang, Y., Uluǧ, A. M., Zimmerman, R. D., & Casey, B. J. (2002). A neural basis for the development of inhibitory control. *Developmental Science*, *5*(4). https://doi.org/10.1111/1467-7687.00235

Eriksen, B. A., & Eriksen, C. W. (1974). Effects of noise letters upon the identification of a target letter in a nonsearch task. *Perception & Psychophysics*, *16*(1), 143–149. https://doi.org/10.3758/BF03203267

Herrero, L., & Carriedo, N. (2018). Differences in updating processes between musicians and non-musicians from late childhood to adolescence. *Learning and Individual Differences*, *61*(10), 188–195. https://doi.org/10.1016/j.lindif.2017.12.006

Herrero, L., & Carriedo, N. (2019). The contributions of updating in working memory sub-processes for sight-reading music beyond age and practice effects. *Frontiers in Psychology*, *10*(JAN). https://doi.org/10.3389/fpsyg.2019.00090

Huizinga, M., Dolan, C. V., & van der Molen, M. W. (2006). Age-related change in executive function: Developmental trends and a latent variable analysis. *Neuropsychologia*, *44*(11), 2017–2036. https://doi.org/10.1016/j.neuropsychologia.2006.01.010

Iglesias-Sarmiento, V., Carriedo, N., Rodríguez-Villagra, O. A., & Pérez, L. (2023). Executive functioning skills and (low) math achievement in primary and secondary school. *Journal of Experimental Child Psychology*, *235*(105715), 1–25. https://doi.org/10.1016/j.jecp.2023.105715

Johnstone, S. J., Pleffer, C. B., Barry, R. J., Clarke, A. R., & Smith, J. L. (2005). Development of inhibitory processing during the Go/NoGo task: A behavioral and event-related potential study of children and adults. *Journal of Psychophysiology*, *19*(1), 11–23. https://doi.org/10.1027/0269-8803.19.1.11

Lechuga, M. T., Moreno, V., Pelegrina, S., Gómez-Ariza, C. J., & Bajo, M. T. (2006). Age differences in memory control: Evidence from updating and retrieval-practice tasks. *Acta Psychologica*, *123*(3), 279–298. https://doi.org/10.1016/j.actpsy.2006.01.006

Leon-Carrion, J., García-Orza, J., & Pérez-Santamaría, F. J. (2004). Development of the inhibitory component of the executive functions in children and adolescents. *International Journal of Neuroscience*, *114*(10), 1291–1311. https://doi.org/10.1080/00207450490476066

Logan, G. D., & Cowan, W. B. (1984). On the ability to inhibit thought and action. *Psychological Review*, *91*, 295–327. https://psycnet.apa.org/record/1994-97487-005

MacLeod, C. M. (2006). The Stroop Task in Cognitive Research. In *Cognitive methods and their application to clinical research.* (pp. 17–40). https://doi.org/10.1037/10870-002

Mayr, S., & Buchner, A. (2007). Negative priming as a memory phenomenon: A review of 20 years of negative priming research. *Journal of Psychology*, *215*(1), 35–51. https://doi.org/10.1027/0044-3409.215.1.35

Mondloch, C. J., Geldart, S., Maurer, D., & de Schonen, S. (2003). Developmental changes in the processing of hierarchical shapes continue into adolescence. *Journal of Experimental Child Psychology*, *84*(1), 20–40. https://doi.org/10.1016/S0022-0965(02)00161-3

Montoro, P. R., Luna, D., & Humphreys, G. W. (2011). Density, connectedness and attentional capture in hierarchical patterns: Evidence from simultanagnosia. *Cortex*, *47*(6), 706–714. https://doi.org/10.1016/j.cortex.2010.05.007

Munro, S., Chau, C., Gazarian, K., & Diamond, A. (2006). Dramatically larger flanker effects (6-fold elevation). *Cognitive Neuroscience Society Annual Meeting*.

Naglieri, J. A., & Das, S. (1997). *Das-Naglieri cognitive assessment system*. Riverside Publishing.

Navon, D. (1977). Forest before trees: The precedence of global features in visual perception. *Cognitive Psychology*, *9*(3), 353–383. https://doi.org/10.1016/0010-0285(77)90012-3

Palladino, P., Cornoldi, C., De Beni, R., & Pazzaglia, F. (2001). Working memory and updating processes in reading comprehension. *Memory and Cognition*, *29*(2), 344–354. https://doi.org/10.3758/BF03194929

Pritchard, V. E., & Neumann, E. (2004). Negative priming effects in children engaged in nonspatial tasks: evidence for early development of an intact inhibitory mechanism. *Developmental Psychology*, *40*(2), 191–203. https://doi.org/10.1037/0012-1649.40.2.191

Rueda, M. R., Fan, J., McCandliss, B. D., Halparin, J. D., Gruber, D. B., Lercari, L. P., & Posner, M. I. (2004). Development of attentional networks in childhood. *Neuropsychologia*, *42*(8), 1029–1040. https://doi.org/10.1016/j.neuropsychologia.2003.12.012

Rueda, M. R., Posner, M. I., & Rothbart, M. K. (2005). The development of executive attention: Contributions to the emergence of self-regulation. *Developmental Neuropsychology*, *28*(2), 573–594. https://doi.org/10.1207/s15326942dn2802_2

Simonds, J., Kieras, J. E., Rueda, M. R., & Rothbart, M. K. (2007). Effortful control, executive attention, and emotional regulation in 7-10-year-old children. *Cognitive Development*, *22*(4), 474–488. https://doi.org/10.1016/j.cogdev.2007.08.009

Snodgrass, J. G., & Vanderwart, M. (1980). A standardized set of 260 pictures: Norms for name agreement, image agreement, familiarity, and visual complexity. *Journal of Experimental Psychology: Human Learning and Memory*, *6*(2), 174–215. https://doi.org/10.1037/0278-7393.6.2.174

St Clair-Thompson, H. L., & Gathercole, S. E. (2006). Executive functions and achievements in school: Shifting, updating, inhibition, and working memory. *Quarterly Journal of Experimental Psychology*, *59*(4), 745–759. https://doi.org/10.1080/17470210500162854

Stroop, J. R. (1935). Studies of interference in serial verbal reactions. *Journal of Experimental Psychology*, *18*(6), 643–662. https://doi.org/10.1037/h0054651

Tamnes, C. K., Østby, Y., Walhovd, K. B., Westlye, L. T., Due-Tønnessen, P., & Fjell, A. M. (2010). Neuroanatomical correlates of executive functions in children and adolescents: A magnetic resonance imaging (MRI) study of cortical thickness. *Neuropsychologia*, *48*(9), 2496–2508. https://doi.org/10.1016/j.neuropsychologia.2010.04.024

Tipper, S. P. (1985). The Negative Priming Effect: Inhibitory Priming By Ignored Objects. *The Quartely Journal of Experimental Psychology*, *37A*, 571–590.

Van Den Wildenberg, W. P. M., & Van Der Molen, M. W. (2004). Additive factors analysis of inhibitory processing in the stop-signal paradigm. *Brain and Cognition*, *56*(2 SPEC. ISS.), 253–266. https://doi.org/10.1016/j.bandc.2004.06.006

Verbruggen, F., Logan, G. D., & Stevens, M. A. (2008). STOP-IT: Windows executable software for the stop-signal paradigm. *Behavior Research Methods*, *40*(2), 479–483. https://doi.org/10.3758/BRM.40.2.479

Waszak, F., Li, S. C., & Hommel, B. (2010). The development of attentional networks: Cross-Sectional findings from a life span sample. *Developmental Psychology*, *46*(2), 337–349. https://doi.org/10.1037/a0018541

1. Although there is considerable debate regarding whether negative priming is a measure of inhibition or of episodic retrieval (Mayr & Buchner, 2007), the consideration that the negative priming effect is accounted for by inhibitory processes is a widely accepted interpretation (see Rueda et al., 2005). We selected negative priming as a measure of cognitive inhibition based on two different criteria: a) previous research results with children (Pritchard & Neumann, 2004); and b) the empirical correlation found in our sample in pilot studies between negative priming and other inhibition-related measures (.45 with previous list intrusions in updating working memory tasks, .67 with flanker task; .59 with local-global task and - .49 with receptive attention task; all *ps* <.01). [↑](#footnote-ref-2)
